# A worldwide study of subcortical shape as a marker for clinical staging in Parkinson’s disease

**DOI:** 10.1038/s41531-024-00825-9

**Published:** 2024-11-19

**Authors:** Max A. Laansma, Yuji Zhao, Eva M. van Heese, Joanna K. Bright, Conor Owens-Walton, Sarah Al-Bachari, Tim J. Anderson, Francesca Assogna, Tim D. van Balkom, Henk W. Berendse, Fernando Cendes, John C. Dalrymple-Alford, Ines Debove, Michiel F. Dirkx, Jason Druzgal, Hedley C. A. Emsley, Jean-Paul Fouche, Gaëtan Garraux, Rachel P. Guimarães, Rick C. Helmich, Michele Hu, Odile A. van den Heuvel, Dmitry Isaev, Ho-Bin Kim, Johannes C. Klein, Christine Lochner, Corey T. McMillan, Tracy R. Melzer, Benjamin Newman, Laura M. Parkes, Clelia Pellicano, Fabrizio Piras, Toni L. Pitcher, Kathleen L. Poston, Mario Rango, Leticia F. Ribeiro, Cristiane S. Rocha, Christian Rummel, Lucas S. R. Santos, Reinhold Schmidt, Petra Schwingenschuh, Letizia Squarcina, Dan J. Stein, Daniela Vecchio, Chris Vriend, Jiunjie Wang, Daniel Weintraub, Roland Wiest, Clarissa L. Yasuda, Neda Jahanshad, Paul M. Thompson, Ysbrand D. van der Werf, Boris A. Gutman

**Affiliations:** 1 Amsterdam UMC, Department of Anatomy and Neurosciences, Vrije Universiteit Amsterdam, Amsterdam, The Netherlands; 2 Amsterdam Neuroscience, Neurodegeneration, Amsterdam, The Netherlands; 3 Department of Biomedical Engineering, Illinois Institute of Technology, Chicago, IL USA; 4 Social, Genetic and Developmental Psychiatry Centre, Institute of Psychiatry, Psychology and Neuroscience, King’s College London, London, UK; 5 Imaging Genetics Center, Mark and Mary Stevens Neuroimaging and Informatics Institute, Keck School of Medicine, University of Southern California, Marina del Rey, CA USA; 6 Faculty of Health and Medicine, The University of Lancaster, Lancaster, UK; 7 Department of Neurology, Royal Preston Hospital, Preston, UK; 8 Department of Medicine, University of Otago, Christchurch, Christchurch, New Zealand; 9 New Zealand Brain Research Institute, Christchurch, New Zealand; 10 Neurology Department, Te Wahtu Ora—Health New Zealand Waitaha Canterbury, Christchurch, New Zealand; 11 Laboratory of Neuropsychiatry, IRCCS Santa Lucia Foundation, Rome, Italy; 12 Amsterdam UMC, Department Psychiatry, Vrije Universiteit Amsterdam, Amsterdam, The Netherlands; 13 Amsterdam UMC, Department Neurology, Vrije Universiteit Amsterdam, Amsterdam, The Netherlands; 14 Department of Neurology, University of Campinas—UNICAMP, Campinas, Brazil; 15 Brazilian Institute of Neuroscience and Neurotechnology, Campinas, Brazil; 16 School of Psychology, Speech and Hearing, University of Canterbury, Christchurch, New Zealand; 17 Department of Neurology, Inselspital, University of Bern, Bern, Switzerland; 18 Department of Neurology and Center of Expertise for Parkinson & Movement Disorders, Donders Institute for Brain, Cognition and Behaviour, Radboud University Nijmegen Medical Centre, Nijmegen, The Netherlands; 19 Department of Radiology and Medical Imaging, University of Virginia, Charlottesville, VA USA; 20 Lancaster Medical School, Lancaster University, Lancaster, UK; 21 Division of Neuroscience and Experimental Psychology, Faculty of Biology, Medicine and Health, The University of Manchester, Manchester Academic Health Science Centre, Manchester, UK; 22 Department of Psychiatry and Mental Health, University of Cape Town, Cape Town, South Africa; 23 GIGA-CRC in vivo imaging, University of Liège, Liège, Belgium; 24 Department of Neurology, CHU Liège, Liège, Belgium; 25 Department of Neurology and Center of Expertise for Parkinson & Movement Disorders, Donders Institute for Brain, Cognition and Behaviour, Radboud University Medical Centre, Nijmegen, The Netherlands; 26 Centre for Cognitive Neuroimaging, Donders Institute for Brain, Cognition and Behaviour, Radboud University Nijmegen, Nijmegen, The Netherlands; 27 Division of Clinical Neurology, Department of Clinical Neurosciences, Oxford Parkinson’s Disease Centre, Nuffield, University of Oxford, Oxford, UK; 28 Department of Biomedical Engineering, Duke University, Durham, NC USA; 29 Department of Neurology & Neurological Sciences, Stanford University, Palo Alto, CA USA; 30 SA MRC Unit on Risk and Resilience in Mental Disorders, Department of Psychiatry, Stellenbosch University, Cape Town, South Africa; 31 University of Pennsylvania Perelman School of Medicine, Philadelphia, PA USA; 32 Division of Psychology, Communication and Human Neuroscience, School of Health Sciences, Faculty of Biology, Medicine and Health, The University of Manchester, Manchester, UK; 33 Geoffrey Jefferson Brain Research Centre, Manchester Academic Health Science Centre, Northern Care Alliance & University of Manchester, Manchester, UK; 34 Excellence Center for Advanced MR Techniques and Parkinson’s Disease Center, Neurology unit, Fondazione IRCCS Cà Granda Maggiore Policlinico Hospital, University of Milan, Milan, Italy; 35 Department of Neurosciences, Neurology Unit, Fondazione Ca’ Granda, IRCCS, Ospedale Policlinico, Univeristy of Milan, Milano, Italy; 36 Support Center for Advanced Neuroimaging, (SCAN) University Institute of Diagnostic and Interventional Neuroradiology, Inselspital, Bern University Hospital, University of Bern, Bern, Switzerland; 37 Department of Neurology, Medical University of Graz, Graz, Austria; 38 Department of Pathophysiology and Transplantation, University of Milan, Milan, Italy; 39 SA MRC Unit on Risk & Resilience in Mental Disorders, Department of Psychiatry and Neuroscience Institute, University of Cape Town, Cape Town, South Africa; 40 Amsterdam Neuroscience, Brain Imaging, Amsterdam, The Netherlands; 41 Department of Medical Imaging and Radiological Sciences, Chang Gung University, Taoyuan City, Taiwan; 42 Department of Diagnostic Radiology, Chang Gung Memorial Hospital, Keelung Branch, Keelung City, Taiwan; 43 Healthy Ageing Research Center, Chang Gung University, Taoyuan City, Taiwan; 44 Support Center for Advanced Neuroimaging (SCAN), University Institute of Diagnostic and Interventional Neuroradiology, University Hospital Bern, Bern, Switzerland

**Keywords:** Parkinson's disease, Neurodegeneration

## Abstract

Alterations in subcortical brain regions are linked to motor and non-motor symptoms in Parkinson’s disease (PD). However, associations between clinical expression and regional morphological abnormalities of the basal ganglia, thalamus, amygdala and hippocampus are not well established. We analyzed 3D T1-weighted brain MRI and clinical data from 2525 individuals with PD and 1326 controls from 22 global sources in the ENIGMA-PD consortium. We investigated disease effects using mass univariate and multivariate models on the medial thickness of 27,120 vertices of seven bilateral subcortical structures. Shape differences were observed across all Hoehn and Yahr (HY) stages, as well as correlations with motor and cognitive symptoms. Notably, we observed incrementally thinner putamen from HY1, caudate nucleus and amygdala from HY2, hippocampus, nucleus accumbens, and thalamus from HY3, and globus pallidus from HY4–5. Subregions of the thalami were thicker in HY1 and HY2. Largely congruent patterns were associated with a longer time since diagnosis and worse motor symptoms and cognitive performance. Multivariate regression revealed patterns predictive of disease stage. These cross-sectional findings provide new insights into PD subcortical degeneration by demonstrating patterns of disease stage-specific morphology, largely consistent with ongoing degeneration.

## Introduction

Alterations in subcortical brain regions play a crucial role in a wide range of clinical domains known to be affected in Parkinson’s disease (PD), including motor, cognitive, emotional, and autonomic functioning^1^. It remains to be fully elucidated, however, how and to what extent localized morphological differences in the basal ganglia, thalamus, amygdala, and hippocampus contribute to the clinical manifestation of PD in vivo.

Shape analysis quantifies local inward and outward variations of the gray matter surface boundaries, complementing standard global volumetry as it can identify subtle regional differences that may not affect total volume. Considering the complex organization of subcortical brain regions, with each nucleus consisting of functionally specialized subdivisions, subcortical shape analysis can provide insights into local vulnerability to disease. An overview of prior findings using shape analysis to study PD is provided in Table S1 in Supplement^2–25^. The majority of case-control studies reported subregional alterations of the putamen and caudate nucleus in PD^2,4,5,8,12,23,25^, while several studies also demonstrated shape abnormalities in the globus pallidus^3,6,12^, hippocampus^6,8^, nucleus accumbens^7^, and thalamus^7,12,19^. The use of predictive modeling to distinguish PD patients from controls based on shape features has been explored; predictive modeling applications in smaller datasets have shown moderate to high classification performance^3,20,21,24^.

While these findings show promise, the reported effects across studies in terms of location, direction, and size vary substantially, which hampers a clear understanding of disease patterns. The inconsistencies may be partly explained by the small sample sizes of individual studies, differences between study sample characteristics, the method quantifying morphometric shape, and the regions of interest selected. Machine learning studies in particular are highly susceptible to overfitting^26^. To address these limitations, it is essential to conduct a well-powered study on a large PD sample that transcends geographical and cultural boundaries, utilizing harmonized processing methods.

Here we present findings from the largest international collaborative analysis of subcortical shape in PD to date, using a combination of mass univariate group comparisons, correlations, and interpretable machine-learning approaches. We aimed to elucidate region-specific morphology patterns and associations with clinical measures. We intended to apply predictive models, not to reach high classification for diagnostic purposes, but rather to better understand disease-stage-related patterns of morphology using a multivariate approach, expanding on our previous work detailing global subcortical volumetry in PD^27^. In line with our prior work, we expected overall thinner subcortical regions in PD compared to controls, except for increased thalamic thickness in mild stages of the disease^27^.

## Results

### Full sample

Data flow for each analysis is depicted in Fig. S1 and a summary of demographic and clinical measures for the PD and control group per source is provided in Table 1 and per cohort in Table S2 in the Supplement. There was a significant difference in age (*D* = 0.12, *p* < 0.001) and sex (*χ*^*2*^(1, *n* = 3851) = 39.3, *p* < 0.001) between the PD and control group.

**Table 1:** Demographics complete sample

			*N*		*N* females		Age ± SD		Time since diagnosis ± SD		MoCA ± SD		MDS-UPDRS3 OFF ± SD	
Site	*N* cohorts	All, (%)	HC	PD	HC	PD	HC	PD	HC	PD	HC	PD	HC	PD
Amsterdam	3	295 (7.7)	74	221	29	85	58.97 ± 9.95	63.17 ± 9.59	NA	3.58 ± 3.95	28.23 ± 1.48	26.20 ± 2.17	NA	28.59 ± 12.74
Bern	2	106 (2.8)	51	55	27	29	62.45 ± 9.92	62.76 ± 10.19	NA	12.36 ± 4.42	NA	23.00 ± 5.66	NA	39.56 ± 12.90
Campinas	1	240 (6.2)	132	108	81	36	58.88 ± 7.77	59.84 ± 10.27	NA	7.33 ± 6.44	NA	NA	NA	NA
Cape Town	1	17 (0.4)	7	10	3	2	66.57 ± 5.68	66.30 ± 5.91	NA	7.12 ± 3.68	26.29 ± 1.89	25.60 ± 3.50	NA	NA
Chang Gung	1	550 (14)	223	327	120	139	60.95 ± 7.28	60.09 ± 9.63	NA	8.70 ± 6.33	NA	NA	NA	28.19 ± 16.93
Charlottesville	3	179 (4.6)		179	NA	45	NA	64.31 ± 8.87	NA	9.25 ± 4.62	NA	24.54 ± 3.63	NA	37.19 ± 10.69
Christchurch	1	263 (6.8)	53	210	18	56	69.13 ± 8.14	69.45 ± 7.77	NA	5.77 ± 5.59	27.06 ± 2.13	23.58 ± 4.18	NA	31.15 ± 17.35
Donders	1	82 (2.1)	23	59	11	26	62.65 ± 10.29	60.81 ± 10.07	NA	4.42 ± 3.79	NA	NA	NA	32.98 ± 15.63
Graz	2	250 (6.5)	125	125	34	34	63.58 ± 10.17	63.58 ± 10.15	NA	4.60 ± 4.98	NA	NA	NA	28.60 ± 19.28
Liege	2	151 (3.9)	76	75	36	31	65.26 ± 6.85	66.48 ± 7.60	NA	6.47 ± 4.55	NA	NA	NA	17.67 ± 9.87
Milan	1	73 (1.9)	25	48	15	15	53.48 ± 8.80	57.54 ± 7.53	NA	11.09 ± 3.55	NA	NA	NA	27.40 ± 11.23
Neurocon^a^	1	42 (1.1)	15	27	12	10	66.73 ± 11.74	68.70 ± 10.55	NA	NA	NA	NA	NA	28.33 ± 9.27
NW-England	2	89 (2.3)	43	46	19	10	68.79 ± 7.29	68.43 ± 8.08	NA	7.55 ± 5.01	27.63 ± 2.19	25.23 ± 3.95	NA	NA
ON Japan^a^	1	45 (1.2)	15	30	8	17	63.33 ± 5.25	67.57 ± 6.81	NA	NA	NA	NA	NA	NA
Oxford	1	181 (4.7)	66	115	23	41	65.95 ± 8.67	63.96 ± 10.17	NA	2.29 ± 1.58	27.36 ± 2.04	26.39 ± 2.79	NA	28.56 ± 13.69
Pennsylvania	1	122 (3.2)	11	111	6	35	70.09 ± 5.86	66.45 ± 7.87	NA	7.35 ± 5.48	NA	25.50 ± 3.30	NA	NA
PPMI^a^	21	504 (13)	159	345	58	120	60.40 ± 11.45	61.67 ± 9.67	NA	0.58 ± 0.56	28.26 ± 1.11	27.15 ± 2.26	NA	20.29 ± 8.59
Rome SLF	1	367 (9.5)	127	240	51	88	36.61 ± 10.56	62.90 ± 10.15	NA	4.96 ± 4.15	NA	NA	NA	16.67 ± 10.67
Stanford	2	213 (5.5)	63	150	39	61	65.53 ± 8.42	68.14 ± 8.68	NA	5.26 ± 4.02	26.95 ± 2.03	25.60 ± 4.27	NA	34.86 ± 12.20
Tao Wu^a^	1	39 (1.0)	20	19	8	9	64.75 ± 5.58	65.00 ± 4.45	NA	5.32 ± 4.00	NA	NA	NA	NA
Udall^a^	1	43 (1.1)	18	25	11	7	62.55 ± 9.97	66.15 ± 10.00	NA	8.93 ± 4.86	27.83 ± 1.47	26.40 ± 2.12	NA	NA
**T**otal	50	3851	1326	2525	609 (46%)	896 (35%)	60.00 ± 12.20	63.69 ± 9.75	NA	5.59 ± 5.43	27.72 ± 1.78	25.69 ± 3.52	NA	28.25 ± 14.39

*N* sample size, *sd* standard deviation, *MoCA* Montreal Cognitive Assessment, *MDS-UPDRS3* Movement Disorders Society sponsored revision of the Unified Parkinson’s Disease Rating Scale part 3, *HC* healthy controls, *PD* Parkinson’s disease.

^a^Open dataset.

### Mass univariate analysis: case-control

**Fig. 1:**
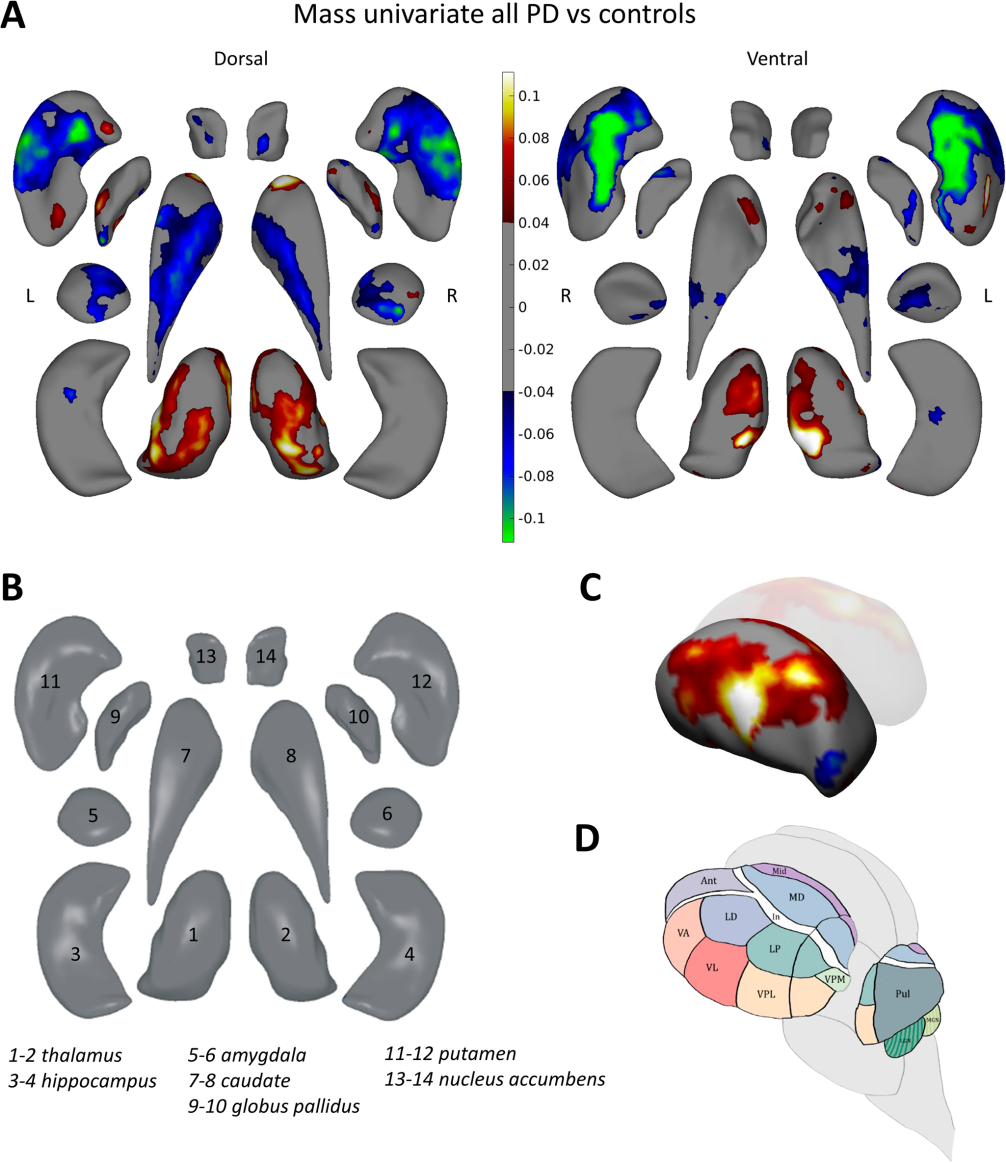
Mass univariate analysis: significant vertex-wise differences in thickness between the full PD group and controls. **A** Effect map projected onto the subcortical regions showing the PD versus control group comparison. Positive *b*-values indicate that regions are thicker and negative *b*-values indicate that regions are thinner in PD compared to controls. The model is corrected for age, sex, intracranial volume and cohort. **B** A dissection of the fourteen subcortical structures of interest in this study to guide the interpretation of panel (**A**). **C** Thalamus effect maps showing group differences and **D** anatomical drawing of the thalamus and its subnuclei displayed approximately in the same angle for interpretation purposes. L left hemisphere, R right hemisphere, Ant anterior nucleus, In intralaminar nuclei, LD lateral dorsal nucleus, LGN lateral geniculate nucleus, LP lateral posterior nucleus, MD mediodorsal nucleus, MGN medial geniculate nucleus, Mid midline nuclei, Pul pulvinar nucleus, VA ventral anterior nucleus, VL ventral lateral nucleus, VPL ventral posterior lateral nucleus, VPM ventral posterior medial nucleus.

The majority of structures were regionally thinner in PD, with the largest differences in the putamen ([% significant of all vertices, peak beta value] left: 42.3%, −0.17; right: 49.2%, −0.18). The thalami, caudate nuclei, globus pallidus, putamen, and left amygdala were regionally thicker, with the largest differences in the thalami (left: 32.4%, 0.13; right 32.4%, 0.13; Fig. 1A–D and Tables S3 and S4 in the Supplement). A comparison of age- and sex-matched PD (*n* = 2502, 35% female, age 63.5 ± 9.5 years) and control (*n* = 610) subsample overall aligned with these patterns (Fig. S2A–B in the Supplement).

### Mass univariate analysis: HY stages

A summary of demographic and clinical measures across Hoehn and Yahr (HY) stages is depicted in Table S5 in the Supplement. Mann–Whitney tests revealed significant differences in the time since diagnosis and Montreal Cognitive Assessment (MoCA) score among all HY groups (Fig. S3A, B in the Supplement). The matching procedure selected 887 controls to match the 451 HY1 participants, 1068 controls to match the 1068 HY2 participants, 846 controls to match the 282 HY3 participants, and 680 controls to match the 85 HY45 participants. The control sample partially overlapped across stage analyses (Table S6 in the Supplement). For optimal matching, some HY2 (135), HY3 (1), and HY45 (1) participants were removed from this analysis.

**Fig. 2:**
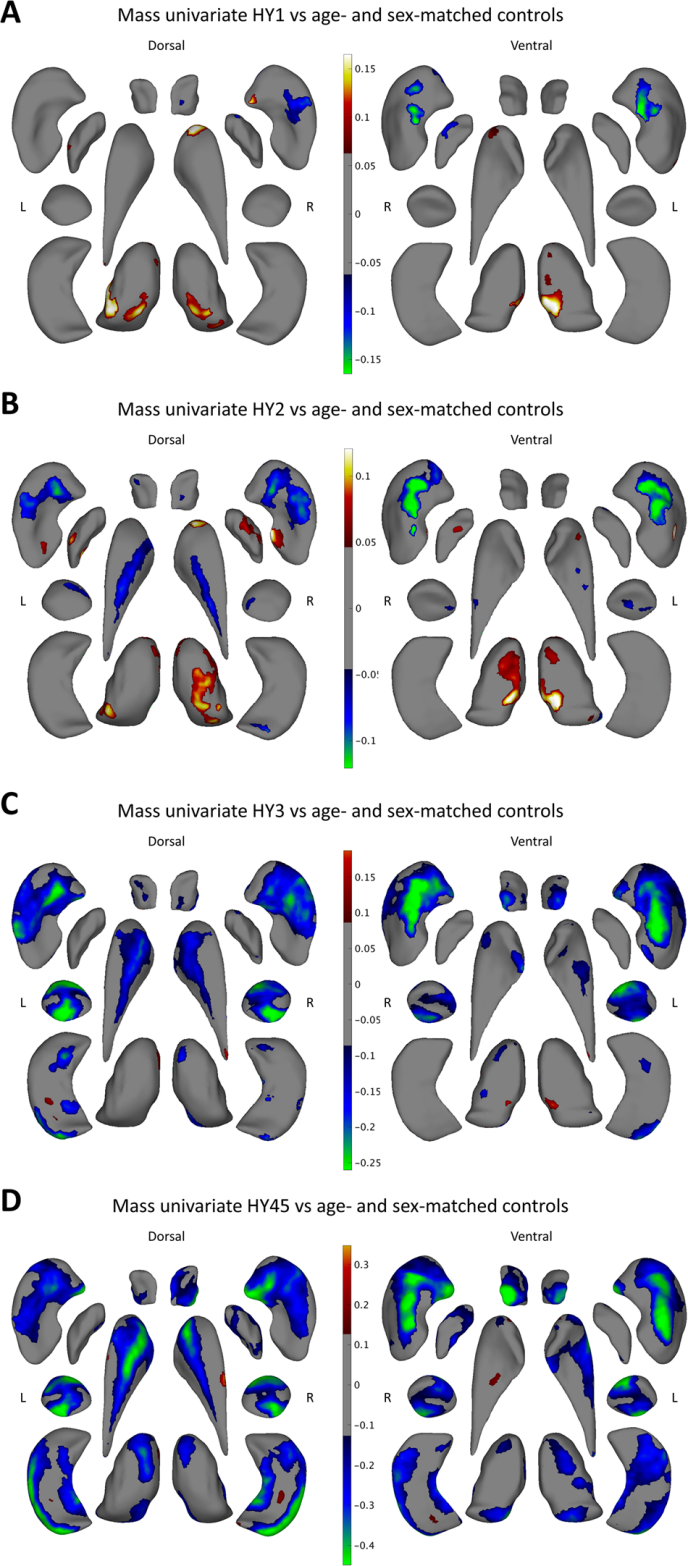
Mass univariate analysis: significant vertex-wise differences in thickness between HY stage and age- and sex-matched controls. **A** HY1, **B** HY2, **C** HY3, and **D** HY45 results are shown in the dorsal and ventral view. Positive *b*-values indicate that regions are thicker in the PD group compared to controls and negative *b*-values indicate that regions are thinner. The models are corrected for intracranial volume, age, sex, and cohort. HY Hoehn and Yahr.

Case-control shape differences were found for all HY stages, notably showing thinner putamen in HY1 ([% significant of all vertices, peak beta value] right: 8.2%, −0.18) and HY2 (left: 16.5%, −0.14; right: 21.8%, −0.17) and thicker bilateral thalamus in HY1 (left: 8.9%, 0.19; right: 19.5%, 0.13) and HY2 (left: 10.5%, 0.15; right: 19.5%, 0.13). Thinner subregions of all structures were identified in HY3 and HY45 (Fig. 2A–D and Table S7A–D in the Supplement). Excluding age and sex as covariates to the models generated comparable patterns (Fig. S2C–F in the Supplement).

Spin permutation tests revealed significant similarities in disease patterns, with breaks in similarity generally between HY2 and HY45. HY1 and HY2 maps were significantly similar for the right putamen (spatial correlation coefficient *r* = 0.52, *p* < 0.001), right caudate nucleus (*r* = 0.48, *p* < 0.001), left globus pallidus (*r* = 0.19, *p* = 0.016), right nucleus accumbens (*r* = 0.53, *p* = 0.006), and thalamus bilaterally (left: *r* = 0.46, *p* = <0.001; right: *r* = 0.39, *p* < 0.001). HY2 and HY3 maps were similar bilaterally for the putamen (left: *r* = 0.53, *p* < 0.001; right: *r* = 0.62, *p* < 0.001) and caudate nucleus (left: *r* = 0.60, *p* < 0.001; right: *r* = 0.28, *p* < 0.001) and the left thalamus (*r* = 0.26, *p* = 0.004). HY3 and HY45 maps were similar for all ROIs (*r*_min_ = 0.46; *r*_max_ = 0.93) except the globus pallidus bilaterally and the left thalamus (see Fig. S4A–C).

### Mass univariate analysis: time since diagnosis, MoCA, and MDS-UPDRS3

A subsample of PD participants had time since diagnosis (*n* = 2350), MoCA (*n* = 1216), and Movement Disorder Society-sponsored revision of the Unified Parkinson’s Disease Rating Scale part 3 (MDS-UPDRS3) (*n* = 1153) scores available. Demographic and clinical characteristics of these subsets are provided in Table S8 in the Supplement. All three variables were moderately correlated with each other (Table S9 in the Supplement).

**Fig. 3:**
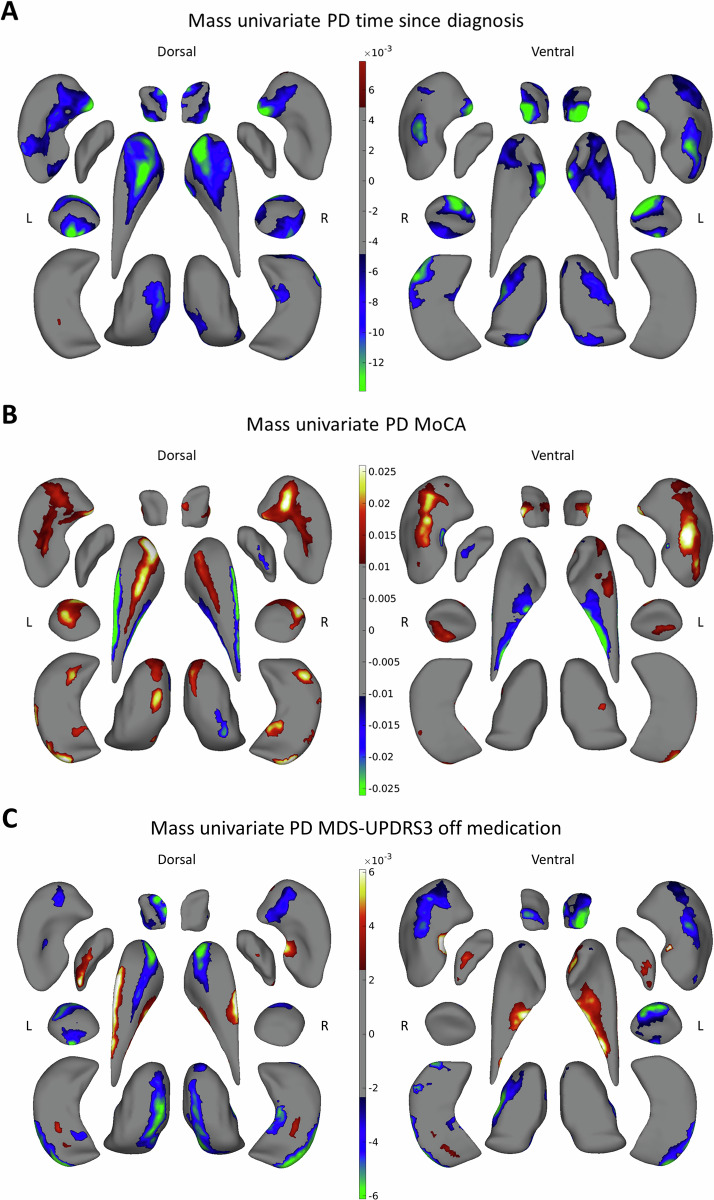
Mass univariate analysis: significant vertex-wise correlation between clinical measures and thickness within the PD group. **A** Time since diagnosis, **B** MoCA, and **C** MDS-UPDRS3 motor score while off medication are shown. Positive *b*-values indicate a positive correlation and negative *b*-values indicate a negative correlation. All regression analyses were corrected for age, sex, and intracranial volume. MoCA Montreal cognitive assessment, MDS-UPDRS3 OFF Movement Disorders Society-sponsored revision of the Unified Parkinson’s disease rating scale part 3 assessed in OFF medication state.

Increasing time since diagnosis was associated with regional thinning in the putamen, caudate nucleus, amygdala, and nucleus accumbens, and to a lesser extent in the thalamus and hippocampus (Fig. 3A and Table S10 in the Supplement). Worse MoCA performance was predominantly associated with regional thinning in the bilateral putamen, amygdala, caudate nucleus, nucleus accumbens, and hippocampus, as well as thicker subregions in the bilateral caudate nucleus, right globus pallidus, and right thalamus (Fig. 3B and Table S11 in the Supplement). Worse MDS-UPDRS3 performance was predominantly associated with regional thinning of the bilateral thalamus, amygdala caudate nucleus, and left nucleus accumbens, as well as thicker subregions of the bilateral caudate nucleus and globus pallidus (Fig. 3C and Table S12 in the Supplement).

### Predictive models for HY classification

**Fig. 4:**
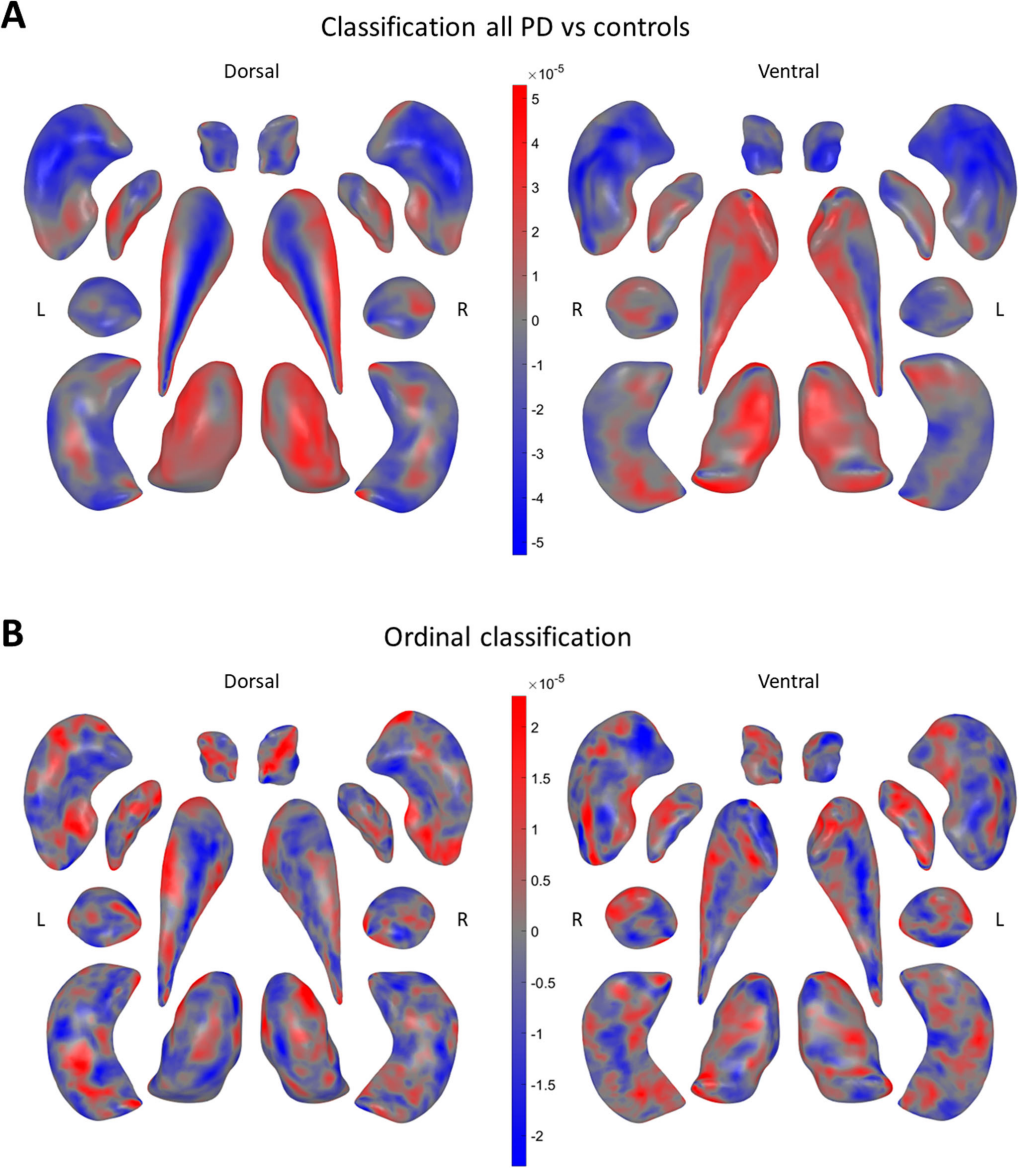
Machine learning: binary and ordinal classification maps. Binary and ordinal classification uses vertex-wise thickness information from all subcortical structures. The color bars represent the learned weights of the classification model, positive values (SD from the learned weights) in red, and negative values in blue. More intense colors indicate a stronger predictive power of the classification. Displayed are the results of **A** the binary classification of people with PD and controls, **B** the ordinal classification of HY1–HY2–HY345.

The patterns of expansion and contraction in the binary classification largely correspond to the patterns shown in the mass univariate model (Fig. 4A), while the ordinal classification shows more dispersed patterns of contractions and expansions (Fig. 4B). Overlaying a hippocampal subfield atlas over the ordinal shape patterns showed greatest atrophy in CA1, CA3, and regions surrounding the hippocampal fissure, and relative sparing of the fimbria and subiculum (Fig. S5A, B in the Supplement).

The TV-L1 regularized logistic regression (Logit-TVL1) model achieved a receiver operating characteristic area-under-the-curve (ROC-AUC) score of 0.65 for the full PD sample compared to controls, 0.61 for HY1 vs HY2 and 0.66 for HY2 vs HY345 classification. The binary classification maps of HY stages contrasted with each other and controls are shown in Figs. S6A–C, S7A–C, and S8A–C in the Supplement. We report on the classification performance between the multi-task classification models (one-against-all vs ordinal logistic regression (Ordit) model) in Table S13 and Fig. S9A–E in the supplement. Additional validity checks on the caudate nucleus revealed no differences in medial curve discrepancy between sites, the PD and control group, and HY disease stages (Fig. S10A–D).

## Discussion

In the largest study on subcortical shape in PD to date, we found local abnormalities of subcortical brain regions in people with PD compared to controls across all disease stages. Each HY increment was characterized by greater impairment in both cognitive and motor domains, as well as greater time since diagnosis, on average, highlighting the close relationship between HY staging and overall clinical progression^28^. Accordingly, the cross-sectional HY stage patterns are in line with the neurodegenerative process that is expected in PD, suggesting progressive thinning of selective subregions of the basal ganglia and limbic regions. This overall aligns with longitudinal findings in mild to more severe PD stages showing the most marked atrophy in the striatum and amygdala^29^.

Largely symmetrical and focal patterns of abnormal shape were found in low stage PD, and more diffuse and pronounced across all structures in higher disease stages. Notably, relatively thinner striatal regions can be observed at the lowest stages. This includes parts of the medial putamen, followed by posterior and anterior putamen regions, and parts of the dorsal caudate nucleus. Later HY stage associations involve the ventral striatum, including the nucleus accumbens. Of note is that the staging pattern aligns with the framework describing the deterioration of the nigrostriatal pathway in PD; subregions of the striatum are shown to not be equally and simultaneously affected; rather, evidence suggests a systematic distribution of pathology across striatal subfields with disease progression, in a similar pattern as presented in the current study^30–34^. Conjecturally, spatial similarity analysis across HY stages suggests that a substantial change in the morphometric correlates of PD progression takes place between HY2 and HY3 across nearly all subcortical regions considered. Only the globus pallidus and the left thalamus have consistent differential patterns of atrophy from HY1 through HY3, with only the HY45-associated shape effects being different from effects in earlier stages.

Cognitive impairment and dementia become increasingly frequent as PD progresses^35^. The implication of first the amygdala and then the hippocampus, accompanied by more selective anterior putamen and anterior-medial dorsal thalamus involvement, fit the expected decline in cognition in this group^30,36^, further emphasized by overlapping subregions that were associated with lower cognitive performance. Overall, the demonstrated regional thinning associated with a longer time since diagnosis agrees with the patterns found in the staging analysis, with a notable difference: the non-linear and non-uniform thalamic pattern across stages can not be captured by the linear regression model, while in turn the implicated subregions largely align with the final HY stage. Regions showing thinning associated with global cognitive and motor impairment largely overlap, highlighting the congruent progression of both domains in PD overall^37^. The bidirectional patterns in the caudate nucleus suggest that the progression of motor and cognitive symptoms is associated with complex shape changes.

Few regions, including parts of the medial dorsal and (ventral) posterolateral thalamus, head of the right caudate nucleus, and lateral globus pallidus, were thicker in mild-stage PD, and did not exhibit thinning even in higher stages. This would suggest that distinct subregions may be temporarily enlarged and then normalized during the disease process, or may be larger premorbidly. The clustering of the mild-stage participants based on shared thalamic shape features revealed no distinct clinical profiles of the identified clusters, suggesting that the variation in thalamic size is not explained by global motor or cognitive severity, see Fig. S11 and Table S14A–D. Possibly, more specific symptom domains are associated with focal enlargement. There is supporting evidence that thalamic hypertrophy may occur in early PD^38^, and could possibly be linked to increased connectivity of thalamic subdivisions that are involved in basal ganglia-thalamocortical and cerebellothalamic circuitry responsible for controlling movement, including the ventral anterior and ventral lateral territories, respectively^39,40^. Evidence suggests that increased functional connectivity of the globus pallidus interna and putamen with the cerebellothalamic pathway is likely involved in the pathophysiology of PD tremor^39^. The continuous activation of the motor system in PD, as well as compensatory homeostasis by remaining cells, may result in a temporary increase in tissue size; a model supported by observations that depletion of striatal dopamine is associated with both striatopallidal hyperactivity^41^ and enlargements of striatopallidal synaptic boutons^42^. The demonstrated relation between greater motor impairment and selectively thicker putamen, caudate nucleus, and globus pallidus territories in this study, and similar findings from a recent study^43^, further support this notion. Other thalamic subdivisions that are among the most densely innervated by dopamine, including the midline limbic nuclei and mediodorsal and lateral posterior association nuclei^44^, seem to be predominantly implicated in mild PD. The pattern agrees with histopathological studies showing that the largest concentrations of Lewy body pathology are found in limbic territories of the thalamus, with the sensory-motor subdivisions being relatively spared^45,46^.

The difference in model maps between binary classification and ordinal HY staging suggests that the morphometric correlates of PD do not vary linearly with disease progression. While regions such as the dorsal caudate nucleus and medial putamen are consistently important in disease prediction, other regions have a more nuanced course. In particular, the dorsal thalamic subregion sees initial expansion (consistent with the univariate analysis), followed by compression in the Ordit model (which excluded control participants). While the binary and ordinal models both work by producing simple linear combinations of local morphometric features, the ordinal model allows for this linear combination to be related non-linearly to a granular measure of disease severity with the addition of only a few more parameters. Critically, this way Ordit strikes a balance between anatomical interpretability and accuracy in severity prediction.

In contrast to univariate analysis, a negatively-weighted region of a subcortical boundary in Ordit may not necessarily imply focal atrophy. While such an interpretation may well be correct, it is also possible that such a region experiences an expansion that is highly correlated with, but reduced relative to, another region with more marked gray matter contraction. In this sense, multivariate maps must be observed as a whole rather than the sum of individual parts^47^. It is precisely this “whole-picture” representation of disease effects—rather than the power to make diagnostic predictions—that captures the value of a multivariate linear shape classifier in the context of PD imaging.

A number of limitations should be discussed. First, the cross-sectional nature of this study complicates making inferences on disease progression. In spite of this, it is striking that the morphometric and clinical patterns in separate PD groups are correlated and strongly incremental, in agreement with progressive degeneration. Secondly, subsamples were available for the analysis due to missing data for some cohorts. While we are aware this may challenge the exchangeability of findings between analyses, we emphasize the unprecedentedly large samples that remained available, enabling the detection of subtle disease patterns that can not be adequately assessed in smaller, underpowered studies. Thirdly, the number of available participants across HY stages was imbalanced. The fewer participants in HY4 and HY5 necessitated combining participants at stage HY3 and higher into one group for the purposes of ordinal regression. This is, however, mitigated by the clinical intuition that the greatest qualitative jump in symptom severity occurs between HY2 and HY3. Fourthly, the level of striatal morphometric asymmetry is possibly dependent on body asymmetry of motor symptom severity, more pronounced in early than late PD stages^48^, which we could not investigate here, but aim to include in future analyses. Finally, the shape method provides fine-grained information on surface deformations, but it does not capture the underlying structure. This reduces the translatability to the subnucleus level, especially in structurally complex brain regions such as the thalamus.

The findings of this unprecedented large study offer new insights into patterns of subcortical degeneration and associations with symptom domains in PD. Notably, the relation between clinical staging and shape alterations aligns with the progressive nature of PD.

## Methods

### Participants

The data in this study were acquired between September 2016 and January 2022 and subsequently pooled by the ENIGMA-PD working group. A total of 3851 3D T1-weighted brain MR images, of 2525 individuals with PD (35% female, age 63.69 ± 9.75 years [mean ± SD]) and 1326 control participants (46% female, 60.00 ± 12.20 years), were included from 22 different sources, resulting in 50 cohorts with distinct scanning and clinical testing environments (Fig. S12). MR images were collected and processed by the individual source institutions; the output of the processing pipeline was uploaded to a central repository for statistical analysis. Clinical characteristics such as HY stage^49^, time since diagnosis, MoCA scores^50^, and scores from the UPDRS3^51^ and the MDS-UPDRS3^52^ obtained in the OFF state were requested for all PD participants and, if available, for controls. HY stage was used as a measure of disease severity and ranged from HY1 to HY5. Participants who were allocated to stages HY1.5 or HY2.5, according to the modified HY classification, were regrouped into HY2. Similarly, smaller groups of PD participants with the most severe stages were merged to increase statistical power, resulting in HY45 for mass univariate analyses and HY345 for the machine learning experiments. Nearest neighbor-matching using the *MatchIt* package in R was performed to create subsamples of age- and sex-matched controls for each HY PD group^53^. Cohort-specific inclusion and exclusion criteria are summarized in Table S2 in the Supplement.

### Imaging acquisition and subcortical shape analysis

T1-weighted MRI scans were collected according to local MRI protocols (Table S15 in the Supplement). All scans were processed using the automated ENIGMA-shape pipeline including FreeSurfer v5.3 recon-all and subcortical parcellation tools^54–59^, together with Matlab and R scripts to perform statistical analysis and visualization (http://enigma.ini.usc.edu/ongoing/enigma-shape-analysis)^60,61^. For each participant, the pipeline extracted a surface mesh of seven regions of interest (thalamus, caudate nucleus, putamen, globus pallidus, hippocampus, amygdala, and nucleus accumbens, unilaterally) representing the outer boundaries of the region. A measure of radial distance was used to examine regional shape deformations across participants, which is computed as the distance from the surface to the medial curve, a smooth curve that is fitted through the approximate center of each structure. This distance is further referred to as thickness^60,62^. Detailed steps of the ENIGMA-Shape pipeline (Fig. S13) and a second measure of shape morphometry (Figs. S13, S14A–H, and S15A–C and Table S16A–H) are reported in the Supplement.

### Mass univariate statistics

Mass univariate statistics across vertices were computed using the lme4 package with standardized R scripts^63^. Vertex-wise group differences (full sample and HY stages vs controls) in thickness were assessed using linear mixed-effects models with the variable *cohort* as a random intercept. Age, sex, and total intracranial volume were added as nuisance covariates. We additionally tested vertex-wise correlations between thickness and variables time since diagnosis (in years), MoCA score, and (MDS-)UPDRS3 off-medication score. UPDRS3 scores were converted to MDS-UPDRS3 scores using a validated formula^64^. For each vertex, we corrected the *p*-value from the mass univariate analyses for multiple comparisons using searchlight false discovery rate correction, at *q* = 0.05 as further explained in the legend of Fig. S13^65^.

### Permutation tests

Over the course of this study, several related statistical maps were derived modeling disease effects at different stages on deep gray-matter structures. The abundance of spatially distributed statistical tests naturally begs the question: in what ways are the patterns of disease similar over the course of PD progression? To quantify spatial coherence between two different effects, we adapted a previously established approach in neuroimaging known as the “spin test”^66^. Briefly, the idea is to preserve the overall spatial structure of the effect pattern while randomly shifting the pattern position with respect to the other effect in the pair. In this way, we can bootstrap a null distribution of spatial correlations between two effect maps—independently in any given brain region—and compare it to the observed correlation. In the case where effects are mapped to a surface of spherical topology, the shift can be accomplished by a spherical rotation leading to the term “spin test”. Here, we generated 10,000 random rotations for each test, using a quaternion representation. The strength of evidence that the patterns are indeed correlated more strongly than by chance can then be measured in the usual frequentist way: the *p*-value is the proportion of times that the absolute value of the observed correlation is greater than the absolute value of the null distribution draws.

### Multivariate predictive models

Due to the unfavorable sample-to-feature ratio in neuroimaging data, effective regularization is critical to train interpretable models that generalize well outside of the training samples. Here, we used a logistic regression (logit) model with “Structured Sparsity”, in which the linear vertex loadings are sparse (using the “L1” norm) and spatially cohesive (using the “Total Variation” or “TV” norm). This combination has been shown to substantially improve model interpretability^67^. Logit-TVL1 has been widely used in functional and diffusion MR brain imaging^67,68^, and more recently for regression and spectral analysis of mesh-based data^69^.

The case-control binary classification was performed by training the Logit-TVL1 on vertex-wise thickness across all structures. Hyper-parameters weighing the relative importance of the TV and L1 terms were optimized using a grid search with 4-fold cross-validation (CV). For assessment, ROC-AUC scores were computed using a “nested” 4-fold-CV approach, which eliminated much of the uncertainty resulting from selecting a single random independent validation subset. Our methods for imputation of missing data and accounting imbalanced groups are described in the legend of Fig. S13.

To identify a multivariate signature of disease progression across all stages using HY stages as the target variable, we used the natural extension of the Logit classification, the Ordit. Briefly, Ordit models consider HY stages to be ordered without assuming any specific functional relationship—such as a linear dependence—between stage and biomarker value. The task is then to simultaneously identify a universal linear multivariate stage progression model and *K* − 1 thresholds *θ*_1_ < *θ*_2_ < *θ*_3_ < … < *θ*_*K−*1_, where K is the number of stages considered. As with the binary models, we used Structured Sparsity regularization. We applied our ordinal classifier using three HY stage classes (HY1, HY2, and HY345). We followed the same hyperparameter tuning and cross-validation approach as with binary classifiers, replacing AUC with a balanced F1 score as performance criteria.

### Inclusion and ethics statement

Within the PD working group of the ENIGMA consortium, we strive for inclusivity by allowing researchers from around the globe to participate in large-scale and impactful investigations. We actively encourage participation from low-income countries and aim to uncover biological insights that transcend borders, race, and socioeconomic status differences. This research was conducted in accordance with the World Medical Association’s Declaration of Helsinki. Approval from the respective institutional review boards and written consent from participants were obtained at each source institution: Amsterdam—Amsterdam I: Medisch Ethische Toetsingscommissie VU Medisch Centrum approval #ID2018.198. Amsterdam II: COGTIPS, METc VUmc #NL58750.029.16 (2016.543). Amsterdam III: Medisch Ethische Toetsingscommissie VU Medisch Centrum approval #ID2018.198. Bern—BE I–II: approved by the Cantonal Ethics Committee (CEC) #2016-00369. Cape Town—Cape Town Stellenbosch: approved by IRBs at Stellenbosch University (IRB reference number: M07/05/019) and the University of Cape Town (IRB reference number: 261/2007). Chang Gung—CGU: Chang Gung Medical Foundation Institutional Review Board #202001592B0. Charlottesville—Charlottesville I–III: University of Virginia Institutional Review Board for Health Science Research #16778. Christchurch: Southern Health and Disability Ethics Committee of the New Zealand Ministry of Health #URB/09/08/037/AM07. Donders—Donders Radboud: METC Oost-Nederland #2014-123 and CMO Regio Arnhem, Nijmegen NL47614.091.14. METC 2014/014. Liege—Liege I–II: The Ethics Committee of the University of Liège approved the study. https://www.sciencedirect.com/science/article/pii/S1053811914005102. Milan: Fondazione Ca’ Granda, IRCCS, Policlinico, the ethical committee approved”, authorization granted by Dr. Giuseppe Di Benedetto, General Director, dated November 19, 2008. This authorization is issued under the authority of Legislative Decree 211/2003, Article 2, Paragraph 1.” Determination number: n.172. NW-England—NW-England I–II: NWE, ethics (North West—Preston Research Ethics Committee) IRAS ID #122770 REC reference 13/NW/0295. Oxford—Oxford DISCOVERY: South-Central Oxford Research Ethics Committee #15/SC/0117. Pennsylvania: University of Pennsylvania Institutional Review Board Protocol #820710. Graz—PROMOVE ASPS I–II: Ethics Committee of the Medical University of Graz #21-345-ex 09/10 and IRB: Medical University of Graz PROMOVE 21-345 ex 09/10 ASPSF: 17-088 ex 05/06. Rome—Rome SLF: Fondazione Santa Lucia Local Institutional Review Board. Approval ID #CE/PROG.905. Stanford—Stanford I–II: The MJFF MRI study (IRB-22722) and the ADRC study (IRB-33727). Campinas—UNICAMP: Comitê de Ética de Pesquisa da UNICAMP; approval #CAAE: 45873415.9.0000.5404.

## Supplementary information

Supplementary Information

## Data Availability

Publicly available datasets used in this work include PPMI (ppmi-info.org), OpenNeuro Japan including Udall cohort (openneuro.org/datasets/ds000245/), and Neurocon and Tao Wu’s data set (fcon_1000.projects.nitrc.org/indi/retro/parkinsons.html). Individual ENIGMA-PD sites retain ownership of their MRI scans and only share the anonymized derived data for this analysis. Data are thus not openly available, but researchers are invited to join the ENIGMA-PD Working Group where they can formally request derived data via secondary proposals. Data requests are then considered by the individual site’s principal investigators. If you are interested in joining ENIGMA-PD, please contact enigma-pd@amsterdamumc.nl. For more information please see the working group website: https://enigma.ini.usc.edu/ongoing/enigma-parkinsons/.

## Code availability

The ENIGMA-Shape pipeline with code to perform processing, statistical analysis, and visualization is available here: http://enigma.ini.usc.edu/ongoing/enigma-shape-analysis. The source code for the in-house analysis scripts for the predictive modeling analysis is made available here: https://github.com/LAMCIG/ML-for-surface-data.
