# Supplementary material for: A worldwide study of subcortical shape as a marker for clinical staging in Parkinson’s disease
Source: NPJ Parkinsons Dis. 2024 Nov 19;10:223. doi: 10.1038/s41531-024-00825-9 (PMC11574005; doi:10.1038/s41531-024-00825-9)
Supplement: Supplementary file 1 — Supplementary Information [file 41531_2024_825_MOESM1_ESM.pdf]

Supplemental Online Content

Table S1. Literature overview of studies performing shape analysis in Parkinson's disease between 2012-2022 2
Table S2. Demographics complete sample per cohort 5
Table S3. Comparison complete sample PD vs controls 7
Table S4. Sample size by ROI and number of excluded failed segmentations 7
Table S5. Hoehn & Yahr Stage Characteristics 7
Table S6. MatchIT controls selection and overlap between Hoehn and Yahr stage analyses 8
Table S7A-D. Results HY-control comparison 9
A - HY1 vs controls 9
B - HY2 vs controls 9
C - HY3 vs controls 9
D - HY4-5 vs controls 10
Table S8. Sample characteristics subgroup analysis 11
Table S9. Pearson's correlation tests between clinical variables of interest 11
Table S10. Results time since diagnosis analysis 11
Table S11. Results MoCA analysis 12
Table S12. Results MDS-UPDRS3 OFF analysis 12
Table S13. Machine learning model comparison 13
Table S14A-D. Results k-means clustering on thalamus vertices of HY1 + HY2 participants 13
A - K=2 Sample descriptives 13
B - K=2 contribution of cohorts to cluster in percentages 14
C - K=3 Sample descriptives 15
D - K=3 contribution of cohorts to cluster in percentages 16
Table S15. Cohort characteristics 18
Table S16A-H. Results Jacobian Determinant 23
A - Complete sample PD vs controls 23
B - HY1 vs controls 23
C - HY2 vs controls 23
D - HY3 vs controls 24
E - HY45 vs controls 24
F - Time since diagnosis in PD 25
G - MoCA in PD 25
H - MDS-UPDRS3 Off in PD 26
Figure S1. Flowchart of data inclusion. 27
Figure S2A-F. Mass univariate analysis: significant vertex-wise differences in thickness between people with PD and controls. 28
Figure S3A-B. Group differences in time since diagnosis and MoCA across HY stage. 29
Figure S4A-C. Pattern coherence analysis with spin permutation tests. 30
Figure S5A-B. Hippocampal subregions on the ordinal classification maps. 31
Figure S6A-C. Machine learning binary classification of HY stages versus each other based on the thickness of subcortical structures. 32
Figure S7A-C. Machine learning: binary classification maps for HY stages versus controls. 33
Figure S8A-C. Machine learning: One-against-All classifications of the HY stages. 34
Figure S9A-E. Machine learning: model comparison One-against-All and Ordit. 35
Figure S10A-D. Caudate nucleus medial curve discrepancies along its longitudinal axis. 36
Figure S11A-C. K-means clustering: determining optimal k 38
Figure S12. World map of the ENIGMA-Parkinson's Disease Datasets. 40
Figure S13. The ENIGMA-Shape Pipeline. 41
Figure S14A-H. Mass univariate analysis: significant vertex-wise differences in Jacobian determinant between people with PD and controls. 42
Figure S15A-C. Comparison of log Jacobian versus thickness effects. 43
Supplementary References (cited in this supplement) 44

Table S1. Literature overview of studies performing shape analysis in Parkinson's disease between 2012-2022

Study	Shape analysis approach	Study population	Key shape deviations	Comments
Baggio et al 2015	Case-control comparison	62 PD<br>compared to<br>31 HC	No significant shape differences	
Caligiuri et al 2016	DAT-SPECT based comparison	23 PD with unilateral abnormal ligand uptake in putamen**<br>compared to<br>30 HC	Shape differences in ipsilateral putamen	Only putamen included as region of interest<br><br>Shape differences did not correlate with clinical variables (age onset, disease duration, UPDRS-ME)
		19 PD with bilateral abnormal ligand uptake in putamen**<br>compared to<br>30 HC	Shape differences in bilateral putamen	
Chung et al 2017	Dementia	74 PD with MCI<br>compared to<br>108 PD without MCI	Shape contractions in bilateral thalamus, right caudate, right hippocampus	
D'Cruz et al 2021	PD subtype comparison	12 PD with freezing of gait and 9 PD converting to freezing of gait<br>compared to<br>36 PD without freezing of gait	Shape expansions in bilateral thalamus	
Devignes et al 2021	Dementia	73 PD with MCI<br>compared to<br>41 PD without MCI	Shape differences in caudate nucleus, hippocampus, thalamus	Only caudate nucleus, hippocampus, thalamus included as regions of interest
Garg et al 2015	Case-control comparison	55 PD<br>compared to<br>54 HC	Shape contractions in left thalamus, bilateral caudate nucleus<br><br>Shape expansions in right putamen, bilateral globus pallidus, left thalamus, bilateral caudate	Shape analysis performed in two data sets with overlapping results<br><br>Only caudate nucleus, putamen, thalamus, globus pallidus included as regions of interest<br><br>Shape differences did not correlate with motor symptoms (UPDRS-3)
	Case-control comparison	189 PD<br>compared to<br>137 HC*		
Gazzina et al 2016	Dementia	16 PD with dementia<br>compared to<br>11 PD without dementia	Non-significant shape contractions in bilateral hippocampus, no shape expansions	Shape analysis was performed only in regions of interest that showed significant differences in global volume, i.e., hippocampus (PDD vs PD) and globus pallidus (DLB vs PD)

		16 Lewy Body dementia compared to 11 PD with dementia	Shape differences in globus pallidus	
Gong et al 2020	Excessive daytime sleepiness	252 PD compared to 92 HC	Shape contractions in bilateral caudate, right putamen	
		59 PD with EDS compared to 193 PD without EDS	Shape expansions in right putamen, left globus pallidus	
Hopes et al 2016	PD staging	70 PD (15 de novo, 40 early, 15 advanced) compared to 20 HC	Shape contractions in caudate nucleus, putamen (for both early and advanced compared to HC/de novo and for early compared to advanced)	Only striatum included as region of interest<br><br>The different PD stages are characterized by striatal shape changes
Lee et al 2014	Case-control comparison	49 untreated early-stage PD compared to 53 HC	Shape contractions in putamen	
Mak et al 2014	Dementia	25 PD with MCI compared to 65 PD without MCI	No significant shape differences	
McKeown et al 2008	Case-control comparison	9 PD compared to 10 HC	Shape differences in bilateral thalamus	Only thalamus included as region of interest
Menke et al 2013	Case-control comparison, classification	20 early PD compared to 19 HC	Shape differences in right globus pallidus	Shape differences showed limited accuracy in the discrimination between PD and HC
Nemmi et al 2015	Case-control comparison	21 PD compared to 20 HC	Shape differences in putamen, caudate	Shape differences correlated with motor symptoms
Nyberg et al 2015	Case-control comparison, PD subtype comparison	21 PD compared to 20 HC	Shape expansions in right nucleus accumbens	Shape differences were driven by a subgroup of tremor-dominant PD patients
Peralta et al 2020	Classification	368 early PD<br>180 advanced PD<br>41 prodromal PD<br>177 HC	NA	Only putamen and caudate nucleus included as regions of interest<br><br>Putamen and caudate nucleus were found differentially informative for classification<br><br>Balanced accuracies ranged from 59-85%

Prashanth et al 2017	Classification	427 early PD compared to 208 HC*	Shape differences in striatum	Shape features distinguished the groups with high accuracy (97%)
Rahayel et al 2019	RBD in PD comparison	15 PD with RBD compared to 15 PD without RBD	Shape contractions in putamen	More severe subcortical neurodegeneration was suggested if PD is accompanied by RBD
		15 PD with RBD compared to 41 HC	Shape contractions in basal ganglia, hippocampus	
		15 PD without RBD compared to 41 HC	Shape contractions in globus pallidus, hippocampus	
De Schipper et al 2019	Dementia	14 Lewy Body dementia compared to 62 PD	Shape contractions in bilateral hippocampus	
Sigirli et al 2021	Case-control comparison	23 early-onset PD compared to 23 HC	Shape differences in right putamen	Only putamen included as region of interest
Sivaranjini et al 2021	Classification of four groups	91 PD without MCI<br>25 PD with MCI<br>19 PD with dementia<br>58 HC*	Shape features differences between the four groups	Only nucleus accumbens, amygdala, caudate nucleus, putamen and thalamus included as regions of interest<br><br>High performance of the classifier using morphological features was observed (88.3 - 96.2%)
Sterling et al 2013	Case-control comparison	40 PD compared to 40 HC	Shape contraction in putamen, caudate nucleus	Only striatum included as region of interest
Tanner et al 2017	Case-control comparison	72 PD compared to 48 HC	Shape differences in bilateral putamen, caudate nucleus, hippocampus	
Vervoort et al 2016	Case-control comparison, PD subtype comparison	55 PD (39 PIGD, 16 TD) compared to 19 HC	Shape contraction in left caudate (PD-PIGD vs HC)	Only putamen, caudate, globus pallidus included as regions of interest

*data obtained from the publicly available Parkinson's Progression Markers Initiative (PPMI)

**as observed on DA transporter single-photon emission tomography

Abbreviations: DAT-SPECT, DA transporter single-photon emission tomography; DLB, Lewy Body Dementia; EDS, excessive daytime sleepiness; HC, healthy controls; MCI, mild cognitive impairment; PD, Parkinson's Disease; PDD, Parkinson's Disease Dementia; PIGD, postural instability and gait disorder; TD, tremor dominant; UPDRS-3, Unified Parkinson's Disease Rating Scale, part 3.

Table S2. Demographics complete sample per cohort

		N			N Females		Age ± SD		Time since diagnosis ± SD		MoCA ± SD		MDS-UPDRS3 OFF ± SD	
**Site**	**Cohort**	**All (%)**	**HC**	**PD**	**HC**	**PD**	**HC**	**PD**	**HC**	**PD**	**HC**	**PD**	**HC**	**PD**
Amsterdam	Amsterdam I	120 (3.1)	0	120	NA	48	NA	63.71 ± 10.85	NA	2.13 ± 3.39	NA	NA	NA	NA
	Amsterdam II	113 (2.9)	30	83	12	33	62.53 ± 9.70	63.25 ± 7.19	NA	5.40 ± 3.86	28.23 ± 1.48	26.20 ± 2.17	NA	23.20 ± 11.09
	Amsterdam III	62 (1.6)	44	18	17	4	56.55 ± 9.48	59.22 ± 9.97	NA	NA	NA	NA	NA	37.58 ± 10.18
Bern	BE I	73 (1.9)	21	52	6	27	54.29 ± 9.83	62.94 ± 10.38	NA	12.42 ± 4.29	NA	23.00 ± 5.66	NA	40.03 ± 13.30
	BE II	33 (0.9)	30	3	21	2	68.17 ± 4.59	59.67 ± 6.66	NA	11.33 ± 7.57	NA	NA	NA	35.00 ± 8.19
Cape Town	Cape Town	17 (0.4)	7	10	3	2	66.57 ± 5.68	66.30 ± 5.91	NA	7.12 ± 3.68	26.29 ± 1.89	25.60 ± 3.50	NA	NA
Chang Gung	CGU	550 (14)	223	327	120	139	60.95 ± 7.28	60.09 ± 9.63	NA	8.70 ± 6.33	NA	NA	NA	28.19 ± 16.93
Charlottesville	Charlottesville I	117 (3.0)	0	117	NA	33	NA	63.62 ± 8.49	NA	9.70 ± 5.07	NA	24.86 ± 3.44	NA	37.01 ± 10.30
	Charlottesville II	38 (1.0)	0	38	NA	5	NA	62.29 ± 9.51	NA	8.82 ± 3.66	NA	24.28 ± 3.45	NA	37.42 ± 11.15
	Charlottesville III	24 (0.6)	0	24	NA	7	NA	70.84 ± 6.77	NA	7.73 ± 3.23	NA	23.39 ± 4.60	NA	37.90 ± 13.30
Christchurch	Christchurch	263 (6.8)	53	210	18	56	69.13 ± 8.14	69.45 ± 7.77	NA	5.77 ± 5.59	27.06 ± 2.13	23.58 ± 4.18	NA	31.15 ± 17.35
Donders	Donders	82 (2.1)	23	59	11	26	62.65 ± 10.29	60.81 ± 10.07	NA	4.42 ± 3.79	NA	NA	NA	32.98 ± 15.63
Liege	Liege I	63 (1.6)	33	30	15	11	65.85 ± 4.29	65.87 ± 6.61	NA	7.23 ± 5.32	NA	NA	NA	17.67 ± 9.87
	Liege II	88 (2.3)	43	45	21	20	64.80 ± 8.33	66.89 ± 8.24	NA	5.96 ± 3.93	NA	NA	NA	NA
Milan	Milan	73 (1.9)	25	48	15	15	53.48 ± 8.80	57.54 ± 7.53	NA	11.09 ± 3.55	NA	NA	NA	27.40 ± 11.23
Neurocon	Neurocon	42 (1.1)	15	27	12	10	66.73 ± 11.74	68.70 ± 10.55	NA	NA	NA	NA	NA	28.33 ± 9.27
NW-England	NW-England I	27 (0.7)	13	14	5	4	64.62 ± 4.13	65.00 ± 5.67	NA	9.21 ± 6.02	29.00 ± 1.41	26.25 ± 2.93	NA	NA
	NW-England II	62 (1.6)	30	32	14	6	70.60 ± 7.65	69.94 ± 8.58	NA	6.83 ± 4.42	27.03 ± 2.22	24.84 ± 4.25	NA	NA
ON Japan	ON Japan	45 (1.2)	15	30	8	17	63.33 ± 5.25	67.57 ± 6.81	NA	NA	NA	NA	NA	NA
Oxford	Oxford DISCOVERY	181 (4.7)	66	115	23	41	65.95 ± 8.67	63.96 ± 10.17	NA	2.29 ± 1.58	27.36 ± 2.04	26.39 ± 2.79	NA	28.56 ± 13.69
Pennsylvania	Pennsylvania	122 (3.2)	11	111	6	35	70.09 ± 5.86	66.45 ± 7.87	NA	7.35 ± 5.48	NA	25.50 ± 3.30	NA	NA
PPMI	PPMI	504 (13)	159	345	58	120	60.40 ± 11.45	61.67 ± 9.67	NA	0.58 ± 0.56	28.26 ± 1.11	27.15 ± 2.26	NA	20.29 ± 8.59
Graz	PROMOVE ASPS I	227 (5.9)	125	102	34	29	63.58 ± 10.17	63.48 ± 10.25	NA	4.73 ± 4.83	NA	NA	NA	29.04 ± 19.99
	PROMOVE ASPS II	23 (0.6)	0	23	NA	5	NA	64.00 ± 9.90	NA	4.04 ± 5.69	NA	NA	NA	24.67 ± 13.20
Rome SLF	Rome SLF	367 (9.5)	127	240	51	88	36.61 ± 10.56	62.90 ± 10.15	NA	4.96 ± 4.15	NA	NA	NA	16.67 ± 10.67

Stanford	Stanford I	173 (4.5)	44	129	27	52	68.09 ± 6.28	69.24 ± 8.28	NA	5.48 ± 4.14	26.95 ± 2.03	25.33 ± 4.39	NA	36.44 ± 12.12
	Stanford II	40 (1.0)	19	21	12	9	59.61 ± 9.86	61.39 ± 8.16	NA	3.73 ± 2.65	NA	27.19 ± 3.06	NA	25.52 ± 7.78
Tao Wu	Tao Wu	39 (1.0)	20	19	8	9	64.75 ± 5.58	65.00 ± 4.45	NA	5.32 ± 4.00	NA	NA	NA	NA
Udal	Udal	43 (1.1)	18	25	11	7	62.55 ± 9.97	66.15 ± 10.00	NA	8.93 ± 4.86	27.83 ± 1.47	26.40 ± 2.12	NA	NA
Campinas	UNICAMP	240 (6.2)	132	108	81	36	58.88 ± 7.77	59.84 ± 10.27	NA	7.33 ± 6.44	NA	NA	NA	NA
**Total**		**3.851**	**1.326**	**2.525**	**609 (46%)**	**896 (35%)**	**60.00 ± 12.20**	**63.69 ± 9.75**	**NA**	**5.59 ± 5.43**	**27.72 ± 1.78**	**25.69 ± 3.52**	**NA**	**28.25 ± 14.39**

Abbreviations: N, sample size; sd, standard deviation; MoCA, Montreal Cognitive Assessment; MDS-UPDRS3, Movement Disorders Society sponsored revision of the Unified Parkinson's disease rating scale part 3; HC, healthy controls; PD, Parkinson's disease.

Table S3. Comparison complete sample PD vs controls

ROI	n vertices	n (%) significant	n (%) thicker	n (%) thinner	thicker max beta	thinner max beta
L thalamus	2502	1129 (45.1)	1101 (44)	28 (1.1)	0.17	-0.09
L caudate	2502	1074 (42.9)	97 (3.9)	977 (39)	0.11	-0.09
L putamen	2502	1165 (46.6)	106 (4.2)	1059 (42.3)	0.14	-0.17
L pallidum	1254	223 (17.8)	95 (7.6)	128 (10.2)	0.13	-0.11
L hippocampus	2502	42 (1.7)	6 (0.2)	36 (1.4)	0.07	-0.09
L amygdala	1368	361 (26.4)	0 (0)	361 (26.4)	0	-0.08
L accumbens	930	42 (4.5)	2 (0.2)	40 (4.3)	0.13	-0.08
R thalamus	2502	813 (32.5)	811 (32.4)	2 (0.1)	0.13	-0.02
R caudate	2502	592 (23.7)	112 (4.5)	480 (19.2)	0.14	-0.08
R putamen	2502	1239 (49.5)	8 (0.3)	1231 (49.2)	0.1	-0.18
R pallidum	1254	216 (17.2)	65 (5.2)	151 (12)	0.13	-0.09
R hippocampus	2502	4 (0.2)	4 (0.2)	0 (0)	0.04	0
R amygdala	1368	279 (20.4)	9 (0.7)	270 (19.7)	0.13	-0.11
R accumbens	930	58 (6.2)	0 (0)	58 (6.2)	0	-0.08
**Overall**	**27120**	**7237 (23.9)**	**2416 (7.4)**	**4821 (16.5)**	**0.17**	**-0.18**

Corrected for age, sex and intracranial volume as fixed factors, and cohort as random intercept.

Table S4. Sample size by ROI and number of excluded failed segmentations

	Analysis N (%)		Segmentation failed N (%)	
**ROI**	**PD (N=2525)**	**Controls (N=1326)**	**PD**	**Controls**
L thalamus	2429 (96.2)	1288 (97.1)	96 (3.8)	38 (2.9)
R thalamus	2355 (93.3)	1257 (94.8)	170 (6.7)	69 (5.2)
L caudate	2458 (97.3)	1302 (98.2)	67 (2.7)	24 (1.8)
R caudate	2420 (95.8)	1279 (96.5)	105 (4.2)	47 (3.5)
L putamen	2442 (96.7)	1293 (97.5)	83 (3.3)	33 (2.5)
R putamen	2443 (96.8)	1296 (97.7)	82 (3.2)	30 (2.3)
L pallidum	2348 (93)	1256 (94.7)	177 (7)	70 (5.3)
R pallidum	2364 (93.6)	1277 (96.3)	161 (6.4)	49 (3.7)
L hippocampus	2354 (93.2)	1260 (95)	171 (6.8)	66 (5)
R hippocampus	2416 (95.7)	1287 (97.1)	109 (4.3)	39 (2.9)
L amygdala	2455 (97.2)	1289 (97.2)	70 (2.8)	37 (2.8)
R amygdala	2451 (97.1)	1301 (98.1)	74 (2.9)	25 (1.9)
L accumbens	2399 (95)	1268 (95.6)	126 (5)	58 (4.4)
R accumbens	2370 (93.9)	1275 (96.2)	155 (6.1)	51 (3.8)

Abbreviations: ROI; Region Of Interest, PD; Parkinson's Disease, L; Left, R; Right.

Table S5. Hoehn & Yahr Stage Characteristics

Characteristic	N	**HY1**, N = 451	**HY2**, N = 1,203	**HY3**, N = 283	**HY4-5**, N = 86
**Age**	2.023	59.54 ± 9.86	64.55 ± 9.06	65.80 ± 10.20	67.38 ± 9.78
***n* (%) Female**	2.023	177 (39%)	401 (33%)	117 (41%)	37 (43%)

**Time since diagnosis**	1.953	2.31 ± 2.54 *	4.99 ± 4.86 *	8.86 ± 6.22 *	13.99 ± 5.64 *
n missing		5	60	3	2
**MoCA**	1.069	27.19 ± 2.32 *	26.02 ± 3.14 *	24.36 ± 3.85 *	19.79 ± 5.21 *
n missing		211	558	132	53
**MDS-UPDRS3 OFF**	1.028	15.29 ± 6.86 *	28.70 ± 11.08 *	39.61 ± 14.77 *	55.08 ± 12.73 *
n missing		150	634	173	38

n (%); Mean ± SD; * Significantly different from other Hoehn & Yahr stage groups on Mann-Whitney test at $p < 0.001$

Table S6. MatchIT controls selection and overlap between Hoehn and Yahr stage analyses

Overlap controls	HY1 (n = 887)	HY2 (n = 1068)	HY3 (n = 846)	HY4-5 (n = 680)
HY1	NA	773	593	456
HY2	773	NA	792	617
HY3	593	792	NA	580
HY4-5	456	617	580	NA

Abbreviations: HY; Hoen and Yahr.

Table S7A-D. Results HY-control comparison

Corrected for age, sex and intracranial volume as fixed factors, and cohort as random intercept.

A - HY1 vs controls

ROI	n vertices	n (%) significant	n (%) thicker	n (%) thinner	thicker max beta	thinner max beta
L thalamus	2502	222 (8.9)	222 (8.9)	0 (0)	0.19	0
R thalamus	2502	96 (3.8)	96 (3.8)	0 (0)	0.15	0
L caudate	2502	1 (0)	1 (0)	0 (0)	0.12	0
R caudate	2502	47 (1.9)	47 (1.9)	0 (0)	0.2	0
L putamen	2502	120 (4.8)	0 (0)	120 (4.8)	0	-0.2
R putamen	2502	205 (8.2)	0 (0)	205 (8.2)	0	-0.18
L pallidum	1254	9 (0.7)	9 (0.7)	0 (0)	0.14	0
R pallidum	1254	50 (4)	0 (0)	50 (4)	0	-0.12
L hippocampus	2502	0 (0)	0 (0)	0 (0)	0	0
R hippocampus	2502	0 (0)	0 (0)	0 (0)	0	0
L amygdala	1368	0 (0)	0 (0)	0 (0)	0	0
R amygdala	1368	0 (0)	0 (0)	0 (0)	0	0
L accumbens	930	0 (0)	0 (0)	0 (0)	0	0
R accumbens	930	7 (0.8)	0 (0)	7 (0.8)	0	-0.1
**Overall**	**27120**	**757 (2.4)**	**375 (1.1)**	**382 (1.3)**	**0.2**	**-0.2**

B - HY2 vs controls

ROI	n vertices	n (%) significant	n (%) thicker	n (%) thinner	thicker max beta	thinner max beta
L thalamus	2502	291 (11.6)	262 (10.5)	29 (1.2)	0.15	-0.13
R thalamus	2502	488 (19.5)	488 (19.5)	0 (0)	0.13	0
L caudate	2502	301 (12)	20 (0.8)	281 (11.2)	0.08	-0.09
R caudate	2502	221 (8.8)	25 (1)	196 (7.8)	0.12	-0.09
L putamen	2502	440 (17.6)	28 (1.1)	412 (16.5)	0.15	-0.14
R putamen	2502	620 (24.8)	74 (3)	546 (21.8)	0.16	-0.17
L pallidum	1254	57 (4.5)	44 (3.5)	13 (1)	0.11	-0.08
R pallidum	1254	93 (7.4)	93 (7.4)	0 (0)	0.1	0
L hippocampus	2502	2 (0.1)	2 (0.1)	0 (0)	0.04	0
R hippocampus	2502	23 (0.9)	3 (0.1)	20 (0.8)	0.07	-0.11
L amygdala	1368	88 (6.4)	0 (0)	88 (6.4)	0	-0.08
R amygdala	1368	39 (2.9)	0 (0)	39 (2.9)	0	-0.07
L accumbens	930	20 (2.2)	0 (0)	20 (2.2)	0	-0.08
R accumbens	930	7 (0.8)	0 (0)	7 (0.8)	0	-0.08
**Overall**	**27120**	**2690 (8.5)**	**1039 (3.4)**	**1651 (5.2)**	**0.16**	**-0.17**

C - HY3 vs controls

ROI	n vertices	n (%) significant	n (%) thicker	n (%) thinner	thicker max beta	thinner max beta
L thalamus	2502	57 (2.3)	53 (2.1)	4 (0.2)	0.19	-0.09
R thalamus	2502	189 (7.6)	9 (0.4)	180 (7.2)	0.17	-0.2

L caudate	2502	590 (23.6)	9 (0.4)	581 (23.2)	0.16	-0.21
R caudate	2502	567 (22.7)	10 (0.4)	557 (22.3)	0.18	-0.22
L putamen	2502	1472 (58.8)	0 (0)	1472 (58.8)	0	-0.3
R putamen	2502	1359 (54.3)	0 (0)	1359 (54.3)	0	-0.35
L pallidum	1254	0 (0)	0 (0)	0 (0)	0	0
R pallidum	1254	24 (1.9)	0 (0)	24 (1.9)	0	-0.28
L hippocampus	2502	402 (16.1)	16 (0.6)	386 (15.4)	0.14	-0.25
R hippocampus	2502	77 (3.1)	0 (0)	77 (3.1)	0	-0.23
L amygdala	1368	1090 (79.7)	0 (0)	1090 (79.7)	0	-0.33
R amygdala	1368	958 (70)	0 (0)	958 (70)	0	-0.31
L accumbens	930	306 (32.9)	0 (0)	306 (32.9)	0	-0.28
R accumbens	930	206 (22.2)	3 (0.3)	203 (21.8)	0.17	-0.28
**Overall**	**27120**	**7297 (28.2)**	**100 (0.3)**	**7197 (27.9)**	**0.19**	**-0.35**

D - HY4-5 vs controls

ROI	n vertices	n (%) significant	n (%) thicker	n (%) thinner	thicker max beta	thinner max beta
L thalamus	2502	739 (29.5)	15 (0.6)	724 (28.9)	0.2	-0.4
R thalamus	2502	507 (20.3)	6 (0.2)	501 (20)	0.12	-0.42
L caudate	2502	1185 (47.4)	16 (0.6)	1169 (46.7)	0.31	-0.5
R caudate	2502	448 (17.9)	46 (1.8)	402 (16.1)	0.35	-0.47
L putamen	2502	1186 (47.4)	3 (0.1)	1183 (47.3)	0.07	-0.52
R putamen	2502	1380 (55.2)	8 (0.3)	1372 (54.8)	0.12	-0.57
L pallidum	1254	47 (3.7)	0 (0)	47 (3.7)	0	-0.53
R pallidum	1254	750 (59.8)	1 (0.1)	749 (59.7)	0.2	-0.53
L hippocampus	2502	1250 (50)	13 (0.5)	1237 (49.4)	0.19	-0.62
R hippocampus	2502	1216 (48.6)	51 (2)	1165 (46.6)	0.22	-0.67
L amygdala	1368	925 (67.6)	0 (0)	925 (67.6)	0	-0.59
R amygdala	1368	872 (63.7)	0 (0)	872 (63.7)	0	-0.49
L accumbens	930	320 (34.4)	1 (0.1)	319 (34.3)	0.2	-0.53
R accumbens	930	772 (83)	0 (0)	772 (83)	0	-0.62
**Overall**	**27120**	**11597 (44.9)**	**160 (0.5)**	**11437 (44.4)**	**0.35**	**-0.67**

Table S8. Sample characteristics subgroup analysis

	Time since diagnosis	MoCA	MDS-UPDRS3 OFF
N	2,350	1,216	1,153
***n* missing**	175	1,309	1,372
**Age**	63.67 ± 9.67	65.14 ± 9.24	62.39 ± 9.71
***n* (%) Female**	829 (35%)	394 (32%)	437 (38%)
**Time since diagnosis**	5.59 ± 5.43	4.57 ± 5.02	5.14 ± 5.76
**MoCA**	25.65 ± 3.53	25.69 ± 3.52	26.28 ± 3.17
**MDS-UPDRS3 OFF**	28.01 ± 14.51	27.05 ± 13.08	28.25 ± 14.39

Abbreviations: MoCA, Montreal Cognitive Assessment; MDS-UPDRS3 OFF, Movement Disorders Society sponsored revision of the Unified Parkinson's disease rating scale part 3 assessed in OFF medication state.

Table S9. Pearson's correlation tests between clinical variables of interest

	MoCA ~ MDS-UPDRS3	MoCA ~ time since diagnosis	MDS-UPDRS3 ~ time since diagnosis
**n**	672	1187	1088
t value	-9.02	-9.88	15.79
**df**	670	1185	1086
p value	**< .001**	**< .001**	**< .001**
**Pearson's r**	-.33	-.28	.43
**conf. int.**	-.39 - -.26	-.33 - -.22	.38 - .48

Pearson's product-moment correlations were tested for subjects with both clinical variables for the test of interest available (MoCA, MDS-UPDRS-III, or time since diagnosis). Abbreviations: MoCA, Montreal Cognitive Assessment; MDS-UPDRS3 OFF, Movement Disorders Society sponsored revision of the Unified Parkinson's disease rating scale part 3 assessed in OFF medication state.

Table S10. Results time since diagnosis analysis

ROI	n vertices	n (%) significant	n (%) positive	n (%) negative	positive beta max	negative beta max
L thalamus	2502	513 (20.5)	0 (0)	513 (20.5)	0	-0.01
R thalamus	2502	598 (23.9)	0 (0)	598 (23.9)	0	-0.01
L caudate	2502	1096 (43.8)	0 (0)	1096 (43.8)	0	-0.02
R caudate	2502	883 (35.3)	0 (0)	883 (35.3)	0	-0.02
L putamen	2502	872 (34.9)	0 (0)	872 (34.9)	0	-0.02
R putamen	2502	371 (14.8)	13 (0.5)	358 (14.3)	0	-0.02
L pallidum	1254	0 (0)	0 (0)	0 (0)	0	0
R pallidum	1254	0 (0)	0 (0)	0 (0)	0	0
L hippocampus	2502	3 (0.1)	3 (0.1)	0 (0)	0.01	0
R hippocampus	2502	322 (12.9)	0 (0)	322 (12.9)	0	-0.01
L amygdala	1368	782 (57.2)	0 (0)	782 (57.2)	0	-0.02
R amygdala	1368	807 (59)	0 (0)	807 (59)	0	-0.02

L accumbens	930	638 (68.6)	0 (0)	638 (68.6)	0	-0.02
R accumbens	930	566 (60.9)	0 (0)	566 (60.9)	0	-0.02
**Overall**	**27120**	**7451 (30.8)**	**16 (0)**	**7435 (30.8)**	**0.01**	**-0.02**

Corrected for age, sex and intracranial volume as fixed factors, and cohort as random intercept.

Table S11. Results MoCA analysis

ROI	n vertices	n (%) significant	n (%) positive	n (%) negative	positive beta max	negative beta max
L thalamus	2502	163 (6.5)	143 (5.7)	20 (0.8)	0.02	-0.02
R thalamus	2502	140 (5.6)	94 (3.8)	46 (1.8)	0.02	-0.02
L caudate	2502	957 (38.2)	436 (17.4)	521 (20.8)	0.03	-0.05
R caudate	2502	662 (26.5)	150 (6)	512 (20.5)	0.02	-0.03
L putamen	2502	718 (28.7)	715 (28.6)	3 (0.1)	0.03	-0.03
R putamen	2502	552 (22.1)	533 (21.3)	19 (0.8)	0.03	-0.02
L pallidum	1254	0 (0)	0 (0)	0 (0)	0	0
R pallidum	1254	55 (4.4)	0 (0)	55 (4.4)	0	-0.02
L hippocampus	2502	200 (8)	200 (8)	0 (0)	0.03	0
R hippocampus	2502	185 (7.4)	185 (7.4)	0 (0)	0.03	0
L amygdala	1368	242 (17.7)	242 (17.7)	0 (0)	0.02	0
R amygdala	1368	317 (23.2)	317 (23.2)	0 (0)	0.03	0
L accumbens	930	95 (10.2)	95 (10.2)	0 (0)	0.02	0
R accumbens	930	165 (17.7)	163 (17.5)	2 (0.2)	0.03	-0.02
**Overall**	**27120**	**4451 (15.4)**	**3273 (11.9)**	**1178 (3.5)**	**0.03**	**-0.05**

Corrected for age, sex and intracranial volume as fixed factors, and cohort as random intercept. Abbreviations: MoCA, Montreal Cognitive Assessment.

Table S12. Results MDS-UPDRS3 OFF analysis

ROI	n vertices	n (%) significant	n (%) positive	n (%) negative	positive beta max	negative beta max
L thalamus	2502	338 (13.5)	0 (0)	338 (13.5)	0	-0.01
R thalamus	2502	617 (24.7)	0 (0)	617 (24.7)	0	-0.01
L caudate	2502	818 (32.7)	601 (24)	217 (8.7)	0.01	-0.01
R caudate	2502	363 (14.5)	201 (8)	162 (6.5)	0.01	-0.01
L putamen	2502	260 (10.4)	20 (0.8)	240 (9.6)	0.01	-0.01
R putamen	2502	432 (17.3)	104 (4.2)	328 (13.1)	0.01	-0.01
L pallidum	1254	163 (13)	163 (13)	0 (0)	0.01	0
R pallidum	1254	44 (3.5)	44 (3.5)	0 (0)	0	0
L hippocampus	2502	276 (11)	17 (0.7)	259 (10.4)	0	-0.01
R hippocampus	2502	438 (17.5)	61 (2.4)	377 (15.1)	0	-0.01
L amygdala	1368	522 (38.2)	0 (0)	522 (38.2)	0	-0.01
R amygdala	1368	57 (4.2)	0 (0)	57 (4.2)	0	-0.01
L accumbens	930	640 (68.8)	0 (0)	640 (68.8)	0	-0.01
R accumbens	930	79 (8.5)	0 (0)	79 (8.5)	0	-0.01
**Overall**	**27120**	**5047 (19.8)**	**1211 (4)**	**3836 (15.8)**	**0.01**	**-0.01**

Corrected for age, sex and intracranial volume as fixed factors, and cohort as random intercept. Abbreviations: MDS-UPDRS3 OFF, Movement Disorders Society sponsored revision of the Unified Parkinson's disease rating scale part 3 assessed in OFF medication state.

Table S13. Machine learning model comparison

		One-against-All	Ordit
F-score	HY1	0.51	0.39
	HY2	0.44	0.58
	HY345	0.42	0.36
Recall	HY1	0.64	0.47
	HY2	0.33	0.55
	HY345	0.59	0.33
Precision	HY1	0.42	0.33
	HY2	0.66	0.62
	HY345	0.33	0.39

Performance metrics for the One-against-All binary classification and Ordit ordinal classification models across Hoehn and Yahr stages. Abbreviations: HY, Hoehn and Yahr.

Table S14A-D. Results k-means clustering on thalamus vertices of HY1 + HY2 participants

Cluster differences were characterized by total volume of the thalamus and other subcortical structures, but not by age, sex, ICV, HY stage, time since diagnosis, age of onset, MDS-UPDRS3, MoCA and/or LEDD.
Abbreviations: HY, Hoehn & Yahr; AO, Age of onset; MoCA, Montreal Cognitive Assessment; MDS-UPDRS3, Movement Disorders Society sponsored revision of the Unified Parkinson's disease rating scale part 3; LEDD, Levodopa equivalent daily dose; ICV, intracranial volume; L, left; R, right.

A - K=2 Sample descriptives

Variable	Cluster 0	Cluster 1	Chi-Squared Test (p-value)		
Sex (% of Females)	38	39	0.736		
HY (% of HY2)	71.8	69.6	0.389		

	Cluster 0 (n=721)		Cluster 1 (n=661)		
Variable	mean	sd	mean	sd	T-Test (p-value)
Age	63.03	9.55	62.78	9.97	0.637
Time since diagnosis	4.33	4.61	4.06	4.13	0.256
AO	58.64	10.37	58.59	10.66	0.936
MDS_UPDRS3	23.46	12.34	23.47	12.31	0.997
MoCA	26.46	3.12	26.28	3.08	0.444
LEDD	538.5	407.16	537.37	420.23	0.974
ICV	1510061.22	192953.7	1507961.14	216367.25	0.849
L thalamus	6837.66	953.1	7782.48	1106.04	**2.82E-57**
R thalamus	6435.01	784.17	7208.3	883.19	**3.45E-59**

L lateral ventricle	14914.79	8168.84	13022.69	8426.27	**2.58E-05**
R lateral ventricle	13611.54	7279	12137.42	8300.33	**0.0005**
L caudate	3364.93	539.87	3491.21	563.05	**2.57E-05**
R caudate	3438.44	545.15	3561.25	560.12	**4.51E-05**
L putamen	4765.49	789.96	4979.87	773.48	**6.57E-07**
R putamen	4572.13	705.84	4798.44	717.3	**5.98E-09**
L pallidum	1570.22	312.53	1643.55	340.03	**4.82E-05**
R pallidum	1556.62	293.38	1622.55	321.5	**8.80E-05**
L hippocampus	3855.58	565.12	4044.41	577.19	**1.80E-09**
R hippocampus	3988.99	561.24	4168.82	564.63	**5.30E-09**
L amygdala	1449.61	261.2	1509.98	251.79	**1.59E-05**
R amygdala	1581.43	288.25	1648.96	289.98	**1.81E-05**
L accumbens	468.07	138.41	496.25	141.81	**0.0002**
R accumbens	475.52	121.65	504.12	125.64	**2.24E-05**

B - K=2 contribution of cohorts to cluster in percentages

	**sample size contribution to total (%)**	
**cohort**	**Cluster 0**	**Cluster 1**
Bern_1	0.8	0.9
CGU_1	12.9	10.9
FSL_1	12.1	12.1
GRAZ_1	5.7	5.6
GRAZ_2	1.4	1.4
Liege_1	1.5	1.5
Liege_2	2.6	3
Milan_1	1.1	1.7
Neurocon_1	1.4	1.7
Oxford_1	6.5	8.6
PDNZ_1	10.7	9.4
PPMI_1	0.7	1.2
PPMI_12	0.8	0.9
PPMI_120	1	1.4
PPMI_154	0.7	0.5
PPMI_18	0.6	0.6
PPMI_196	0.1	0.2
PPMI_23	0.1	0.2
PPMI_28	0.8	0.6
PPMI_289	1	1.1

PPMI_290	1.2	1.4
PPMI_291	0.3	0.6
PPMI_32	0.8	1.5
PPMI_327	0.4	0.5
PPMI_34	3.9	3.2
PPMI_40	0.8	1.1
PPMI_57	0.8	0.8
PPMI_6	0.7	1.2
PPMI_7	1.5	1.2
PPMI_88	1.4	0.9
PPMI_96	1.4	1.2
Penn_1	2.9	2.1
RADBOUD_1	2.6	2.4
Stanford_1	6.5	6.7
Stanford_2	1.4	1.4
Stellenbosch	0.1	0.2
TaoWu_1	1	0.8
UNICAMP_1	3.7	4.5
UOMmain_1	0.7	0.8
UOMpilot_1	0.1	0.2
Udal_1	1.7	1.7
VUMC_2	3.3	2.7

C - K=3 Sample descriptives

				**p-value**					
	**Cluster 0**	**Cluster 1**	**Cluster 2**	**0 vs 1**	**0 vs 2**	**1 vs 2**			
Sex (% F)	37.7	39	39.5	0.747	0.631	0.942			
HY1234 (% HY2)	70.8	68.4	73.4	0.464	0.435	0.174			

	**Cluster 0 (n=681)**		**Cluster 1 (n=367)**		**Cluster 2 (n=334)**		**t-test p-value**		
	**mean**	**sd**	**mean**	**sd**	**mean**	**sd**	**0 vs 1**	**0 vs 2**	**1 vs 2**
Age	63.13	9.71	62.51	10.31	62.9	9.19	0.335	0.712	0.604
Time since diagnosis	4.29	4.49	4.06	4.03	4.17	4.58	0.418	0.694	0.741
AO	58.8	10.44	58.29	10.8	58.61	10.33	0.462	0.788	0.694
MDS_UPDRS3	23.42	12.12	22.83	12.55	24.26	12.49	0.486	0.327	0.153
MoCA	26.48	3.13	26.27	2.86	26.29	3.29	0.434	0.533	0.931

LEDD	555.65	421.96	549.54	444.54	494.52	358.37	0.889	0.137	0.237
ICV	1515173.22	197356.09	1511727.1	231610.08	1493651.64	185667.4	0.8	0.096	0.258
L thalamus	7254.16	967.99	8071.7	1123.57	6489.2	839.6	**1.63E-31**	**1.93E-31**	**1.52E-72**
R thalamus	6772.26	780.01	7441.9	896.33	6167.51	717.05	**3.04E-33**	**6.01E-30**	**1.68E-72**
L lateral ventricle	13505.02	7915.39	13185.29	8934.07	15961.29	8243.05	0.552	**5.88E-06**	**2.52E-05**
R lateral ventricle	12384.35	7248.29	12412.56	8938.74	14513.18	7402.81	0.956	**1.52E-05**	**0.0008**
L caudate	3395.8	536.48	3555.17	588.1	3342.42	528.9	**1.16E-05**	0.139	**8.42E-07**
R caudate	3464.18	569.44	3618.23	537.25	3431.69	526.93	**2.52E-05**	0.388	**5.49E-06**
L putamen	4858	810.11	5016.71	779.5	4724.07	728.39	**0.0027**	**0.012**	**5.82E-07**
R putamen	4680.13	729.03	4845.49	707.6	4498.37	671.56	**0.0005**	**0.0002**	**9.01E-11**
L pallidum	1609.81	322.71	1654.48	349.75	1540.83	302.47	**0.043**	**0.0014**	**8.21E-06**
R pallidum	1600.38	296.91	1628.8	339.47	1517.29	285.19	0.165	**3.19E-05**	**4.66E-06**
L hippocampus	3945.09	541.93	4109.24	611.56	3767.54	562.62	**1.17E-05**	**2.10E-06**	**1.44E-13**
R hippocampus	4093.59	544.59	4214.28	591.97	3882.37	544.02	**0.001**	**1.29E-08**	**8.02E-14**
L amygdala	1476.59	245.75	1538.82	257.47	1415.7	270	**0.0001**	**0.0004**	**1.57E-09**
R amygdala	1603.49	280.32	1679.57	296.89	1561.54	293.35	**4.74E-05**	**0.0298**	**2.31E-07**
L accumbens	479.64	137.8	504.29	148.43	460.29	134.41	**0.0077**	**0.0357**	**5.20E-05**
R accumbens	489.19	126.24	513.59	119.4	462.31	120.6	**0.0026**	**0.0014**	**3.03E-08**

D - K=3 contribution of cohorts to cluster in percentages

	**sample size contribution to total (%)**		
**Cohort**	**Cluster 0**	**Cluster 1**	**Cluster 2**
Bern_1	1.2	0.5	0.6
CGU_1	14.4	10.1	9
FSL_1	8.8	14.2	16.5
GRAZ_1	5.9	5.2	5.7
GRAZ_2	1.5	1.4	1.2
Liege_1	0.9	1.9	2.4
Liege_2	3.7	1.6	2.4
Milan_1	0.9	2.2	1.5
Neurocon_1	1.8	1.4	1.2
Oxford_1	6.2	9	8.7
PDNZ_1	9.5	10.9	10.2
PPMI_1	0.9	0.8	1.2
PPMI_12	0.9	0.8	0.9

PPMI_120	1	1.4	1.2
PPMI_154	0.6	0.5	0.6
PPMI_18	0.9	0.3	0.3
PPMI_196	0.1	0.3	0
PPMI_23	0.3	0	0
PPMI_28	1.2	0.3	0.3
PPMI_289	1	0.8	1.2
PPMI_290	1.5	1.1	1.2
PPMI_291	0.4	0.3	0.6
PPMI_32	1.3	1.1	0.9
PPMI_327	0.4	0.3	0.6
PPMI_34	3.2	4.1	3.6
PPMI_40	1.3	0.5	0.6
PPMI_57	1	0.5	0.6
PPMI_6	1	0.8	0.9
PPMI_7	1.3	1.4	1.5
PPMI_88	1.3	1.1	0.9
PPMI_96	1.2	1.6	1.2
Penn_1	3.4	2.2	1.2
RADBOUD_1	2.8	2.7	1.8
Stanford_1	6.9	6	6.6
Stanford_2	1.2	1.9	1.2
Stellenbosch	0.3	0	0
TaoWu_1	0.9	0.8	0.9
UNICAMP_1	4	4.1	4.5
UOMmain_1	0.7	0.8	0.6
UOMpilot_1	0.1	0	0.3
Udal_1	2.3	1.1	0.9
VUMC_2	1.8	4.1	4.5

Table S15. Cohort characteristics

Site	Cohort	Diagnostic criteria	Time between MRI and clinical assessment	MRI acquisition details	PD	HC
Amsterdam	Amsterdam I	UKBB	Same day	GE Discovery (3T); Sagittal 3-dimensional gradient-echo T1-weighted sequence (256 x 256 matrix; FOV = 25cm; voxel size = 1 x 0.98 x 0.98 mm; TR = 7.8 ms; TE = 3.0 ms; FA = 12°)	Inclusion: consecutive patients seen at the movement disorders outpatient clinic.<br>Exclusion: -	No controls
	Amsterdam II (Cogtips)	UKBB	Same day		Inclusion: Subjective cognitive complaints (PD-CFRS > 3), HY stage < 4.<br>Exclusion: dementia (SAGE <14 or MoCA < 22), drugs or alcohol abuse (CAGE AID > 1), depressive symptoms (BDI > 18), impulse control disorder (ICD criteria interview), psychotic symptoms (SAPS-PD criteria), tumors and significant vascular abnormalities.	Inclusion: sex, age, and education-matched<br>Exclusion: neurological disease, indication of dementia (MoCA < 22), indication of psychotic (SAPS-PD) or depressive disorder (BDI > 18), drugs and/or alcohol abuse, inability to undergo neuropsychological assessment, traumatic brain injury, tumor or vascular abnormalities.
	Amsterdam III	UKBB	Same day		Inclusion: early stage, non-demented PD patients who were not using dopamine replacement therapy.<br>Exclusion: current psychiatric or neurological disorders other than PD, a Beck Depression Inventory (BDI) score >15 and a Mini Mental State Examination (MMSE) score <24.	Inclusion: sex, age, education, and handedness-matched<br>Exclusion: current psychiatric or neurological disorders, a Beck Depression Inventory (BDI) score >15 and a Mini Mental State Examination (MMSE) score <24.
Bern	BE I	UKBB	Within 7 days	Siemens Verio (3T); MDEFT sequence (1mm³ isotropic voxel; TR = 7.92ms, TE = 2.48ms, TI=910ms)	Inclusion: PD and familial forms of typical Parkinsonian syndromes, motor complications of dopaminergic medication that are at least moderately bothersome to the patient.<br>Exclusion: Age > 85 years, surgical or medical contraindications for a deep brain stimulation (DBS)-implantation, severe medical illness, severe personality disorder, dementia (DSM-V criteria and MMSE < 20, current psychosis, ongoing major depression (BDI-II > 23) or depression of any severity with suicidal ideation.	Inclusion: sex and age-matched<br>Exclusion: -
	BE II			Siemens Trio Tim (3T); as above		
Campinas	UNICAMP	UKBB	15.3 days on average (standard deviation=11.1)	Philips Achiva (3T); 3D T1 weighted image acquired on the sagittal plane (FOV of	Inclusion: idiopathic PD, taking antiparkinsonian medications, age > 30 years.	Inclusion: age > 30 years old.<br>Exclusion: clinically significant

				240x240mm; 1mm³ isotropic voxel, TR = 7ms, TE = 3.2ms; FA = 8°)	Exclusion: clinically significant musculoskeletal, cardiovascular, respiratory or other neurological disease.	musculoskeletal, cardiovascular, respiratory or neurological disease.
Cape Town	Cape Town	MDS	15.0 days on average	Siemens Magnetom Skyra (3T); 3D T1 weighted image acquired on the sagittal plane (FOV of 256x256mm; 1mm³ isotropic voxel, TR = 2530, TE = 1.69, TI = 1100, FA = 7°)	Inclusion: diagnosis of PD by neurologist , age > 40 years and <= than 75 years.<br>Exclusion: Any significant medical/physical illness (other than PD), participants with metal prostheses, cardiac pacemakers or metal clips likely to interfere with ability to acquire MR image	Inclusion: no current or lifetime history of any DSM-5 psychiatric disorder<br>Exclusion: Neurological conditions that would preclude completion of neurocognitive tasks, current or lifetime daily psychotropic medication use.
Chang Gung	CGU	NINDS	Within 30 days, except for one participant (45 days)	Siemens Magnetom Trio Tim (3T); T1-weighted images were acquired using an MPRAGE (224×256 matrix; FOV = 224 mm × 256 mm; 1mm³ isotropic voxel; TE = 2.63 ms; TR = 2000 ms, FA = 9°)	Inclusion: diagnosis of probable PD, ability to tolerate treatment discontinuation for 12 hours.<br>Exclusion: major physical illnesses, psychiatric disorders, known brain abnormalities, history of intracranial surgery, pharmacotherapy for more than ten years or treatment with drugs able to cross the blood- brain-barrier (other than those used to treat PD).	Inclusion: aged between 50- 90.<br>Exclusion: same as in PD.
Charlottesville	Charlottesville I-III	PD diagnosis confirmed by neurologist	73.9 days on average	Siemens (3T); Stock MPRAGE. Acquisition parameters vary by scanner protocol.<br>Voxel size varied but did not exceed 1 × 1 × 1.2 mm.	Inclusion: PD diagnosis with a motor symptom that is not (or inconsistently) responsive to oral medication.<br>Exclusion: -	No controls
Christchurch	Christchurch	UKBB	28 days on average (standard deviation=48)	General Electric HDx (3T); SPGR sequence	Inclusion: met the UK Parkinson's Society criteria for PD, motor symptoms present for at least 1 year at study entry.<br>Exclusion: atypical parkinsonian disorder, history of moderate/severe head injury, stroke, early-life learning disability, major psychiatric or medical illness in the previous 6 months, poor English (precluding testing).	Inclusion: -<br>Exclusion: neurological disease/disorder; history of moderate/severe head injury, stroke, early-life learning disability, major psychiatric or medical illness in the previous 6 months, poor English (precluding testing).
Donders	Donders	UKBB	Same day	Siemens Magnetom Trio (3T); 3D T1 weighted image acquired on the sagittal plane (FOV of 256x256mm;	Inclusion: Idiopathic PD, UPDRS tremor-score > 2, dopaminergic therapy with a clear clinical response of non-tremor symptoms (bradykinesia,	Inclusion: same age/gender balance as PD patients<br>Exclusion: Neurological or psychiatric disease, cognitive impairment (MMSE <

				1mm³ isotropic voxel, TR = 2300, TE = 3.03, TI = 1100, FA = 8°)	rigidity), HY stage 1-3.<br>Exclusion: Neurological or psychiatric comorbidity, severe head tremor or dyskinesias, cognitive impairment (MMSE < 26), co-medication associated with elongated QT-time, pregnancy, age < 25 years.	26), medication associated with elongated QT-time, pregnancy, age < 25 years.
Graz	PROMOVE/ ASPS I&II	QSBB	90% same day, maximum of 4 weeks	Siemens Magnetom Trio/Prisma (3T); PD: structural T1-weighted MPRAGE sequence (1mm³ isotropic voxel; TR = 1900ms; TI = 900ms; FA = 9°;<br>+ TE: 2.19ms (101 patients)<br>+ TE: 2.7ms (23 patients)<br>HC: structural T1-weighted MPRAGE sequence (1mm³ isotropic voxel; TR = 1900ms; TE = 2.19ms; TI = 900ms; FA = 9°)	Inclusion: Clinical diagnosis of PD.<br>Exclusion: MMSE <24, secondary parkinsonism, atypical parkinsonian diseases, a history of neuroleptic drugs, structural abnormalities on routine MRI scans or a history of previous stroke.	Inclusion: No history of previous stroke or dementia and a normal neurologic examination.<br>Exclusion: -
Liege	Liege I & II	UKBB	Same day	Siemens Magnetom Allegra (3T); 3D multi-echo fast low angle shot (FLASH) sequence, (256 × 224 matrix; 1mm³ isotropic voxel; TR = 18.7 ms; TE = 2.2-14.7 ms; FA = 20°)	Inclusion: Non-demented PD patients.<br>Exclusion: -	Inclusion: age, sex, and highest achieved education level matched.<br>Exclusion: -
Manchester	NW-England I	UKBB	Same day	Philips Achieva (3T); MPRAGE IR Method (voxel size 0.94 x 0.94 x 1 mm; FOV 240 (AP) x 192 (RL) mm TR=8.4ms, TE=3.9ms, TI=1150ms, FA = 8°)	Inclusion: PD diagnosis without known clinical cardiovascular disease or dementia. No other significant neurological conditions.	Inclusion: age-matched to PD group and without a history of idiopathic PD or clinical CVD, or any other significant neurological condition.
	NW-England II	UKBB	Same day		Inclusion: as above.<br>Exclusion: -	Inclusion: age-matched to PD group and without a history of idiopathic PD or other significant neurological condition.
Milan	Milan	UKBB	Within 1 month	Philips Achieva (3T); 240x240mm matrix; 1mm³ isotropic voxel; FOV = 33.7x24 cm; TR = 9.81ms; TE = 4.6ms; FA = 8°	Inclusion: PD diagnosis.<br>Exclusion: -	Inclusion: -<br>Exclusion: -

NEUROCON	NEUROCON	MDS	Not available	Siemens Avanto (1.5T); MPRAGE IR Method. (voxel size 0.97 x 0.97 x 1mm; TR 1940ms TE 3.08ms)	Inclusion: Early- or moderate stage of PD.<br>Exclusion: -	Inclusion: no history of neurological or psychiatric disease.
ON Japan	ON Japan	UKBB	Not available	Siemens Magnetom Verio (3T); High resolution T1-weighted images (256 × 256 matrix size; FOV = 256 mm; TR = 2.5 s, TE = 2.48 ms)	Inclusion: -<br>Exclusion: history of other neurological or psychiatric disease, focal white matter abnormalities. ACE-R score ≤ 88, psychiatric symptoms (hallucinations, depression etc)	Inclusion: -<br>Exclusion: neurological disease, family history of PD, or hyposmia, and with an ACE-R score > 88 in the study
Oxford	Oxford DISCOVERY	UKBB	108 days on average (standard deviation=104)	Siemens Trio (3T); MPRAGE (1mm³ isotropic voxel, TE = 4.7 ms; TR = 2040 ms; TI ¼ = 900ms; FA: 8°)	Inclusion: PD diagnosis within the past 3.5 years. Full details of criteria are available at: Szewczyk-Krolikowski K et. al. (2013). No atypical features to suggest an alternative diagnosis.<br>Exclusion: secondary parkinsonism due to head trauma or medication use, atypical parkinsonism syndromes (multiple system atrophy, progressive supra nuclear palsy, corticobasal degeneration, dementia with Lewy bodies), documented postural BP drop on standardized measurement or significant urinary symptoms.	Inclusion: controls without blood relatives with PD.
Pennsylvania	Pennsylvania	UKBB	MoCA: 53.7 days on average (standard deviation=67.1)<br><br>HY: 51.4 days on average (standard deviation=65.0)	Siemens Trio/Prisma (3T); 3D MPRAGE Sagittal & Axial (Slice thickness = 1mm; TR = 1620/1800/2300ms; TE = 2.95/3.8/3.09ms; TI = 900/950ms)	Inclusion: clinical diagnosis of PD.<br>Exclusion: -	Inclusion: >40 years of age, MMSE > 27, a negative self-reported history of neurological or psychiatric condition, and MRI safe (e.g., no metal, claustrophobia).<br>Exclusion: -
PPMI	PPMI 1-21	MDS	Same day	Siemens Trio Tim (3T); T1-3D e.g. MPRAGE, SPGR, Sagittal (56 x 256 x 170-200 matrix;<br>Slice thickness = 1.2mm; voxel size 1x1x1.2mm)	Inclusion & Exclusion criteria detailed here: www.ppmi-info.org/wp-content/uploads/2013/02/PPMI-Protocol-AM5-Final-27Nov2012v6-2.pdf.	Inclusion & Exclusion criteria detailed here: www.ppmi-info.org/wp-content/uploads/2013/02/PPMI-Protocol-AM5-Final-27Nov2012v6-2.pdf.
Rome SLF	Rome SLF	MDS	1 day	Siemens Allegra (3T); T1 MDEFT (256x224 matrix; 1mm³ isotropic voxel; TR =	Inclusion: diagnosis of idiopathic, MMSE score>26, no dementia.<br>Exclusion: presence of major non stabilized medical, known or suspected	Inclusion: vision and hearing sufficient for compliance with testing procedures, laboratory values within normal reference intervals, neuropsychological domain

				7.92ms; TE = 2.4ms; FA = 15°)	history of alcoholism, drug dependence and abuse, head trauma, and mental disorders (apart from mood or anxiety disorders, history of neurological diseases other than idiopathic PD, unclear history of chronic dopaminergic treatment responsiveness.	scores above normal cognitive level cutoff scores, corrected for age and educational level.<br>Exclusion: dementia or MCI diagnosis, confirmed by a comprehensive neuropsychological battery, MMSE score<26, presence of major non stabilized medical illnesses, known or suspected history of alcoholism, drug dependence and abuse, head trauma, and mental disorders (apart from mood or anxiety disorders).
Stanford	Stanford	UKBB	Within 3 months	General Electric SIGNA (3T); FSPGR 3D T1 scan	Inclusion: > 20% improvement on MDS-UPDRS part III ON meds compared to OFF meds.<br>Exclusion: -	Inclusion: normal neurological exam and normal neuropsychiatric battery (within 1.5 SD of age- and education- adjusted norms).<br>Exclusion: -
Tao Wu	Tao Wu	MDS	1-2 days	Siemens Magnetom Trio (3T); MPRAGE IR method ($1mm^3$ isotropic voxel; TR 1100ms; TE 3.39ms)	Inclusion: diagnosis of PD based on medical history, physical and neurological examinations, response to levodopa or dopaminergic drugs, and laboratory tests and MRI scans to exclude other diseases.<br>Exclusion: -	Inclusion: -<br>Exclusion: -
Udall	Udall	Not available	Not available	Philips Achieva (3T); sagittal T1-weighted 3D MPRAGE (176 slices, matrix size = 256 × 256, inversion time = 1100 ms, turbo-field echo factor = 225, repetition time = 7.46 ms, echo time = 3.49 ms, flip angle = 7°, shot interval = 2530 ms) with 1 mm isotropic voxels	Inclusion:<br>Exclusion: Potential participants were excluded if they had a history of any primary neurodegenerative disease other than idiopathic PD, brain surgery (including placement of a deep brain stimulator), moderate to severe dyskinesia, significant head trauma, stroke history, severe or unstable cardiovascular disease, contraindications to MRI, or a Montreal Cognitive Assessment score (MoCA) (Nasreddine et al., 2005) lower than 23	Not available

Scanner protocol information for each sample included in analyses. Abbreviations: PD = Parkinson's disease; HC = healthy controls; TR = Repetition Time; TE = Echo time; TI = Inversion time; FOV = field of view; FA = flip angle

Table S16A-H. Results Jacobian Determinant

Corrected for age, sex and intracranial volume as fixed factors, and cohort as random intercept. Abbreviations: MoCA, Montreal Cognitive Assessment; MDS-UPDRS3 OFF, Movement Disorders Society sponsored revision of the Unified Parkinson's disease rating scale part 3 assessed in OFF medication state.

A - Complete sample PD vs controls

ROI	n vertices	n (%) significant	n (%) thicker	n (%) thinner	thicker max beta	thinner max beta
L thalamus	2502	9 (0.4)	9 (0.4)	0 (0)	0.02	0
R thalamus	2502	87 (3.5)	0 (0)	87 (3.5)	0	-0.03
L caudate	2502	606 (24.2)	0 (0)	606 (24.2)	0	-0.04
R caudate	2502	722 (28.9)	87 (3.5)	635 (25.4)	0.02	-0.03
L putamen	2502	323 (12.9)	223 (8.9)	100 (4)	0.04	-0.03
R putamen	2502	365 (14.6)	103 (4.1)	262 (10.5)	0.03	-0.02
L pallidum	1254	631 (25.2)	631 (25.2)	0 (0)	0.03	0
R pallidum	1254	1299 (51.9)	1299 (51.9)	0 (0)	0.04	0
L hippocampus	2502	130 (9.5)	0 (0)	130 (9.5)	0	-0.04
R hippocampus	2502	13 (1)	0 (0)	13 (1)	0	-0.02
L amygdala	1368	475 (37.9)	5 (0.4)	470 (37.5)	0.03	-0.05
R amygdala	1368	585 (46.7)	108 (8.6)	477 (38)	0.04	-0.04
L accumbens	930	186 (20)	0 (0)	186 (20)	0	-0.06
R accumbens	930	348 (37.4)	0 (0)	348 (37.4)	0	-0.06
**Overall**	**27120**	**5779 (22.4)**	**2465 (7.4)**	**3314 (15.1)**	**0.04**	**-0.06**

B - HY1 vs controls

ROI	n vertices	n (%) significant	n (%) thicker	n (%) thinner	thicker max beta	thinner max beta
L thalamus	2502	0 (0)	0 (0)	0 (0)	0	0
R thalamus	2502	0 (0)	0 (0)	0 (0)	0	0
L caudate	2502	33 (1.3)	0 (0)	33 (1.3)	0	-0.03
R caudate	2502	0 (0)	0 (0)	0 (0)	0	0
L putamen	2502	63 (2.5)	63 (2.5)	0 (0)	0.03	0
R putamen	2502	7 (0.3)	7 (0.3)	0 (0)	0.03	0
L pallidum	1254	26 (1)	26 (1)	0 (0)	0.04	0
R pallidum	1254	133 (5.3)	133 (5.3)	0 (0)	0.04	0
L hippocampus	2502	0 (0)	0 (0)	0 (0)	0	0
R hippocampus	2502	0 (0)	0 (0)	0 (0)	0	0
L amygdala	1368	162 (12.9)	0 (0)	162 (12.9)	0	-0.07
R amygdala	1368	131 (10.4)	0 (0)	131 (10.4)	0	-0.05
L accumbens	930	0 (0)	0 (0)	0 (0)	0	0
R accumbens	930	0 (0)	0 (0)	0 (0)	0	0
**Overall**	**27120**	**555 (2.4)**	**229 (0.7)**	**326 (1.8)**	**0.04**	**-0.07**

C - HY2 vs controls

ROI	n vertices	n (%) significant	n (%) thicker	n (%) thinner	thicker max beta	thinner max beta
L thalamus	2502	0 (0)	0 (0)	0 (0)	0	0

R thalamus	2502	36 (1.4)	0 (0)	36 (1.4)	0	-0.03
L caudate	2502	202 (8.1)	0 (0)	202 (8.1)	0	-0.04
R caudate	2502	277 (11.1)	0 (0)	277 (11.1)	0	-0.03
L putamen	2502	29 (1.2)	29 (1.2)	0 (0)	0.03	0
R putamen	2502	63 (2.5)	0 (0)	63 (2.5)	0	-0.02
L pallidum	1254	132 (5.3)	132 (5.3)	0 (0)	0.03	0
R pallidum	1254	585 (23.4)	550 (22)	35 (1.4)	0.03	-0.03
L hippocampus	2502	0 (0)	0 (0)	0 (0)	0	0
R hippocampus	2502	5 (0.4)	0 (0)	5 (0.4)	0	-0.03
L amygdala	1368	12 (1)	12 (1)	0 (0)	0.03	0
R amygdala	1368	74 (5.9)	36 (2.9)	38 (3)	0.04	-0.03
L accumbens	930	177 (19)	0 (0)	177 (19)	0	-0.07
R accumbens	930	305 (32.8)	0 (0)	305 (32.8)	0	-0.08
**Overall**	**27120**	**1897 (8)**	**759 (2.3)**	**1138 (5.7)**	**0.04**	**-0.08**

D - HY3 vs controls

ROI	n vertices	n (%) significant	n (%) thicker	n (%) thinner	thicker max beta	thinner max beta
L thalamus	2502	168 (6.7)	0 (0)	168 (6.7)	0	-0.06
R thalamus	2502	1100 (44)	0 (0)	1100 (44)	0	-0.06
L caudate	2502	1410 (56.4)	0 (0)	1410 (56.4)	0	-0.09
R caudate	2502	1367 (54.6)	0 (0)	1367 (54.6)	0	-0.08
L putamen	2502	562 (22.5)	0 (0)	562 (22.5)	0	-0.05
R putamen	2502	490 (19.6)	0 (0)	490 (19.6)	0	-0.05
L pallidum	1254	330 (13.2)	0 (0)	330 (13.2)	0	-0.04
R pallidum	1254	68 (2.7)	10 (0.4)	58 (2.3)	0.03	-0.03
L hippocampus	2502	802 (58.6)	0 (0)	802 (58.6)	0	-0.11
R hippocampus	2502	1024 (74.9)	0 (0)	1024 (74.9)	0	-0.13
L amygdala	1368	453 (36.1)	0 (0)	453 (36.1)	0	-0.1
R amygdala	1368	325 (25.9)	0 (0)	325 (25.9)	0	-0.1
L accumbens	930	388 (41.7)	0 (0)	388 (41.7)	0	-0.12
R accumbens	930	613 (65.9)	0 (0)	613 (65.9)	0	-0.13
**Overall**	**27120**	**9100 (37.3)**	**10 (< 0.1)**	**9090 (37.3)**	**0.03**	**-0.13**

E - HY45 vs controls

ROI	n vertices	n (%) significant	n (%) thicker	n (%) thinner	thicker max beta	thinner max beta
L thalamus	2502	1355 (54.2)	0 (0)	1355 (54.2)	0	-0.12
R thalamus	2502	1283 (51.3)	0 (0)	1283 (51.3)	0	-0.13
L caudate	2502	1339 (53.5)	0 (0)	1339 (53.5)	0	-0.15
R caudate	2502	1305 (52.2)	22 (0.9)	1283 (51.3)	0.05	-0.17
L putamen	2502	263 (10.5)	107 (4.3)	156 (6.2)	0.11	-0.07
R putamen	2502	474 (18.9)	164 (6.6)	310 (12.4)	0.1	-0.09
L pallidum	1254	496 (19.8)	26 (1)	470 (18.8)	0.06	-0.05
R pallidum	1254	799 (31.9)	11 (0.4)	788 (31.5)	0.05	-0.07

L hippocampus	2502	857 (62.6)	0 (0)	857 (62.6)	0	-0.19
R hippocampus	2502	884 (64.6)	0 (0)	884 (64.6)	0	-0.21
L amygdala	1368	573 (45.7)	0 (0)	573 (45.7)	0	-0.11
R amygdala	1368	220 (17.5)	0 (0)	220 (17.5)	0	-0.11
L accumbens	930	790 (84.9)	0 (0)	790 (84.9)	0	-0.2
R accumbens	930	554 (59.6)	0 (0)	554 (59.6)	0	-0.25
**Overall**	**27120**	**11192 (44.8)**	**330 (0.9)**	**10862 (43.9)**	**0.11**	**-0.25**

F - Time since diagnosis in PD

ROI	n vertices	n (%) significant	n (%) positive	n (%) negative	positive beta max	negative beta max
L thalamus	2502	771 (30.8)	0 (0)	771 (30.8)	0	-0.003
R thalamus	2502	21 (0.8)	0 (0)	21 (0.8)	0	-0.002
L caudate	2502	439 (17.5)	0 (0)	439 (17.5)	0	-0.005
R caudate	2502	1125 (45)	0 (0)	1125 (45)	0	-0.005
L putamen	2502	1135 (45.4)	0 (0)	1135 (45.4)	0	-0.004
R putamen	2502	769 (30.7)	0 (0)	769 (30.7)	0	-0.004
L pallidum	1254	136 (5.4)	0 (0)	136 (5.4)	0	-0.002
R pallidum	1254	263 (10.5)	0 (0)	263 (10.5)	0	-0.002
L hippocampus	2502	889 (65)	0 (0)	889 (65)	0	-0.005
R hippocampus	2502	1050 (76.8)	0 (0)	1050 (76.8)	0	-0.007
L amygdala	1368	0 (0)	0 (0)	0 (0)	0	0
R amygdala	1368	0 (0)	0 (0)	0 (0)	0	0
L accumbens	930	576 (61.9)	0 (0)	576 (61.9)	0	-0.008
R accumbens	930	357 (38.4)	0 (0)	357 (38.4)	0	-0.006
**Overall**	**27120**	**7531 (30.6)**	**0 (0)**	**7531 (30.6)**	**0**	**-0.008**

G - MoCA in PD

ROI	n vertices	n (%) significant	n (%) positive	n (%) negative	positive beta max	negative beta max
L thalamus	2502	307 (12.3)	303 (12.1)	4 (0.2)	0.008	-0.004
R thalamus	2502	378 (15.1)	319 (12.8)	59 (2.4)	0.007	-0.006
L caudate	2502	442 (17.7)	442 (17.7)	0 (0)	0.007	0
R caudate	2502	629 (25.1)	629 (25.1)	0 (0)	0.009	0
L putamen	2502	125 (5)	0 (0)	125 (5)	0	-0.009
R putamen	2502	436 (17.4)	169 (6.8)	267 (10.7)	0.008	-0.008
L pallidum	1254	171 (6.8)	124 (5)	47 (1.9)	0.004	-0.005
R pallidum	1254	273 (10.9)	273 (10.9)	0 (0)	0.007	0
L hippocampus	2502	324 (23.7)	324 (23.7)	0 (0)	0.011	0
R hippocampus	2502	457 (33.4)	457 (33.4)	0 (0)	0.011	0
L amygdala	1368	0 (0)	0 (0)	0 (0)	0	0
R amygdala	1368	0 (0)	0 (0)	0 (0)	0	0
L accumbens	930	83 (8.9)	83 (8.9)	0 (0)	0.011	0
R accumbens	930	254 (27.3)	254 (27.3)	0 (0)	0.013	0
**Overall**	**27120**	**3879 (14.5)**	**3377 (13.1)**	**502 (1.4)**	**0.013**	**-0.009**

H - MDS-UPDRS3 Off in PD

ROI	n vertices	n (%) significant	n (%) positive	n (%) negative	positive beta max	negative beta max
L thalamus	2502	843 (33.7)	0 (0)	843 (33.7)	0	-0.002
R thalamus	2502	512 (20.5)	0 (0)	512 (20.5)	0	-0.001
L caudate	2502	472 (18.9)	233 (9.3)	239 (9.6)	0.002	-0.002
R caudate	2502	280 (11.2)	59 (2.4)	221 (8.8)	< 0.001	-0.002
L putamen	2502	42 (1.7)	14 (0.6)	28 (1.1)	0.001	< -0.001
R putamen	2502	715 (28.6)	715 (28.6)	0 (0)	0.002	0
L pallidum	1254	130 (5.2)	0 (0)	130 (5.2)	0	-0.001
R pallidum	1254	332 (13.3)	0 (0)	332 (13.3)	0	-0.001
L hippocampus	2502	271 (19.8)	0 (0)	271 (19.8)	0	-0.002
R hippocampus	2502	689 (50.4)	0 (0)	689 (50.4)	0	-0.002
L amygdala	1368	129 (10.3)	129 (10.3)	0 (0)	0.001	0
R amygdala	1368	100 (8)	100 (8)	0 (0)	0.002	0
L accumbens	930	81 (8.7)	0 (0)	81 (8.7)	0	-0.002
R accumbens	930	596 (64.1)	0 (0)	596 (64.1)	0	-0.004
**Overall**	**27120**	**5192 (21)**	**1250 (4.2)**	**3942 (16.8)**	**0.002**	**-0.004**

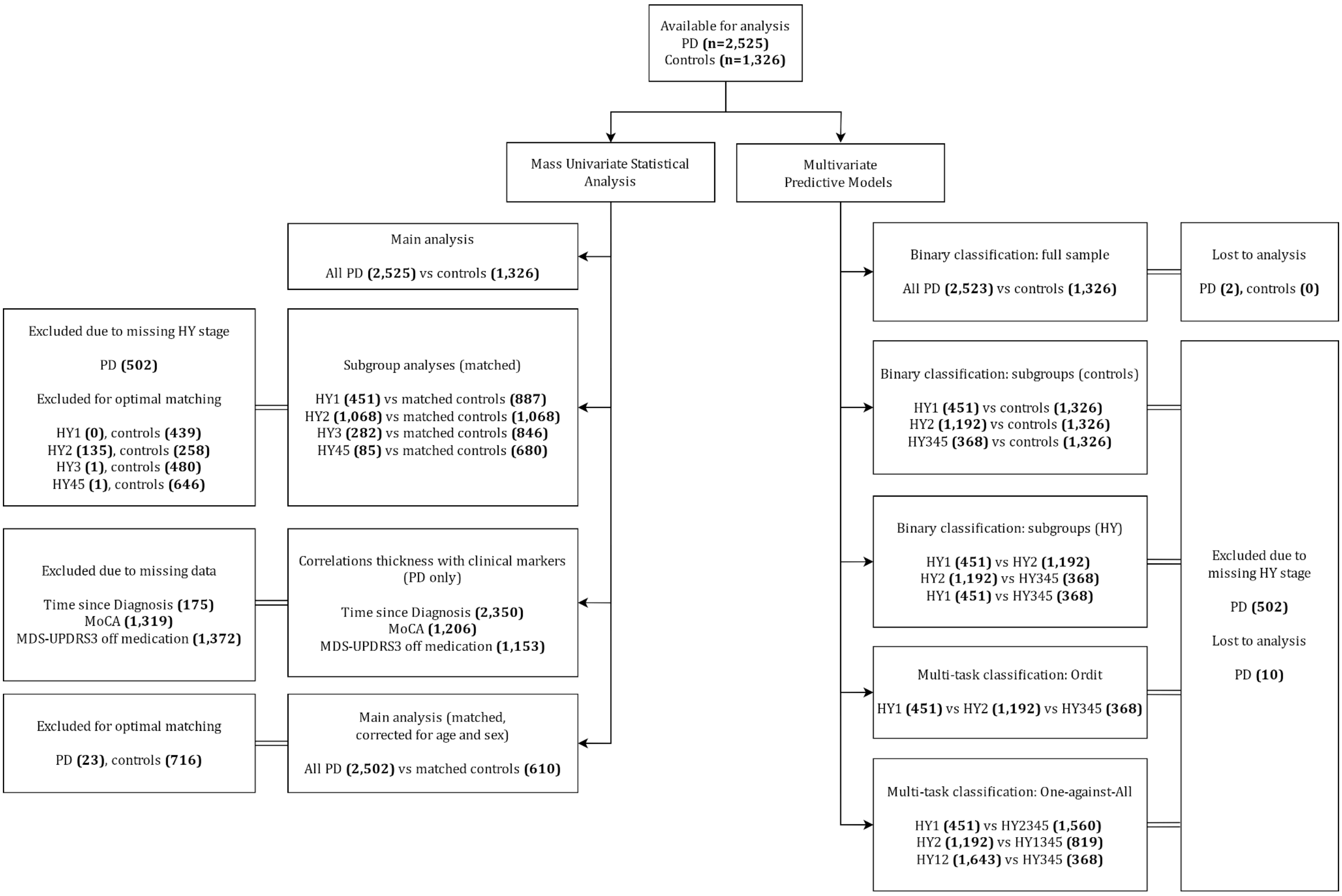

Figure S1. Flowchart of data inclusion.

Schematic overview of available datasets for each analysis, categorized by 'Mass Univariate Statistical Analysis' and 'Multivariate Predictive Models'. For the univariate analyses, we performed group comparison and correlation analysis on radial thickness. For the multivariate analyses, binary classification and multi-task classification were performed. Abbreviations: PD, Parkinson's disease; MoCA, Montreal Cognitive Assessment; MDS-UPDRS3, Movement Disorder Society sponsored revision of the Unified Parkinson's Disease Rating Scale part 3; HY, Hoehn and Yahr.

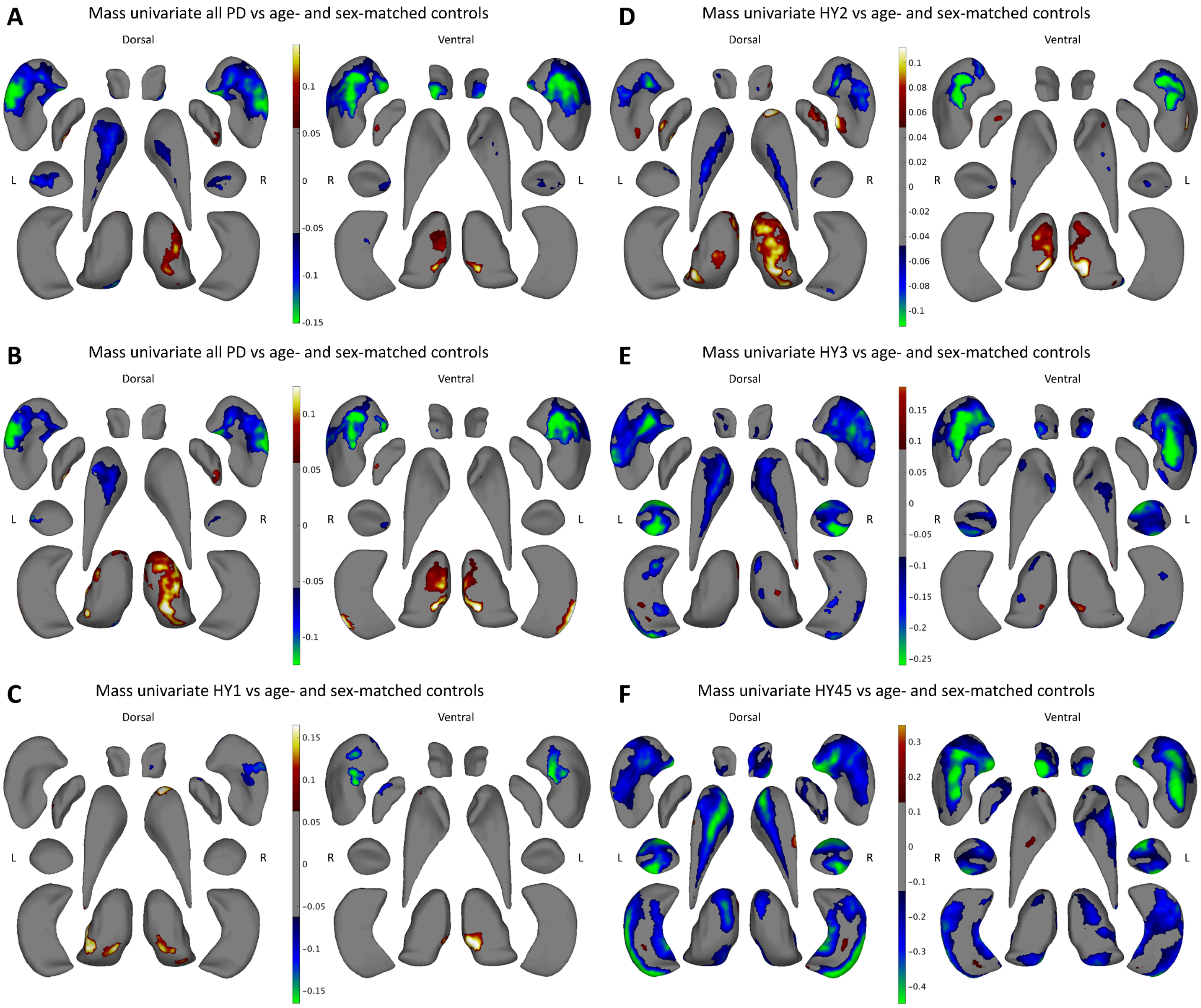

Figure S2A-F. Mass univariate analysis: significant vertex-wise differences in thickness between people with PD and controls.

Effect maps are projected onto the subcortical regions showing (**A-B**) the PD versus controls and (**C-F**) subgroup comparisons. Positive b-values indicate that regions are thicker, negative b-values indicate that regions are thinner in PD compared to controls. All groups are sex- and age-matched (**A-F**). The models are corrected for intracranial volume and cohort in all panels, and additionally corrected for age and sex in panel **B**.

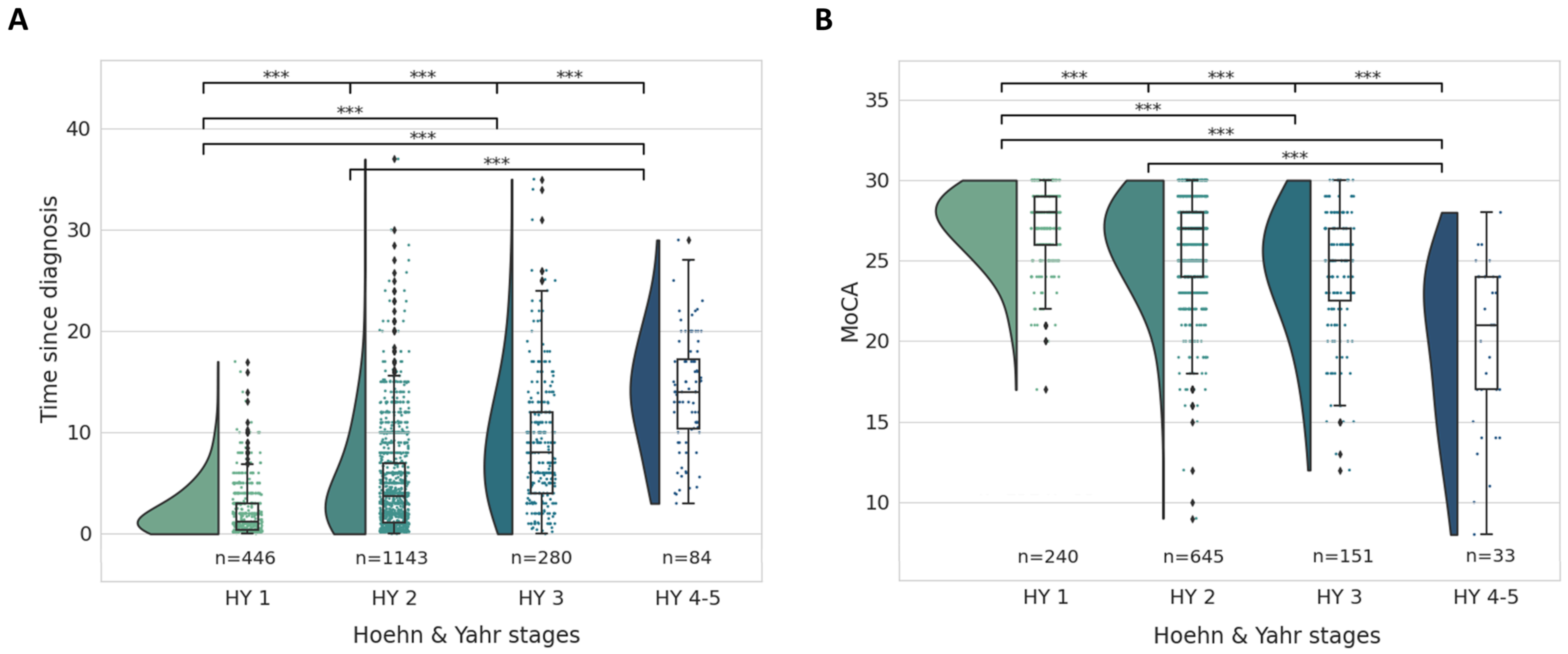

Figure S3A-B. Group differences in time since diagnosis and MoCA across HY stage.

(**A**) Time since diagnosis (in years) and (**B**) MoCA scores are shown for HY stages 1 to 4-5. Sample sizes are shown below the raincloud plots. *** indicates $p < .001$.

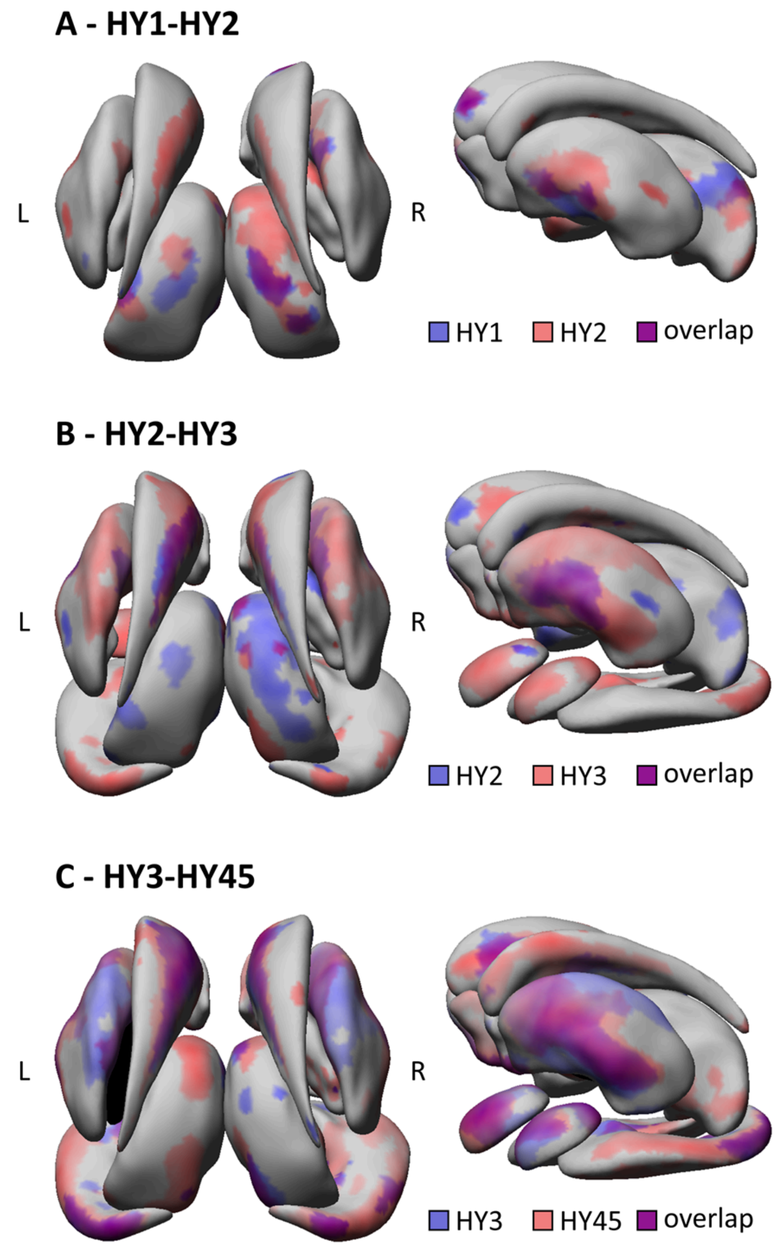

Figure S4A-C. Pattern coherence analysis with spin permutation tests.
Each panel shows the effect maps of two case-control mass univariate analyses are projected onto the subcortical regions to highlight overlapping patterns between the stages (purple), regardless of the direction of the effects. (**A**) HY1 vs controls (blue) and HY2 vs controls (pink), (**B**) HY2 vs controls (blue) and HY3 vs controls (pink), (**C**) HY3 vs controls (blue) and HY45 vs controls (pink).

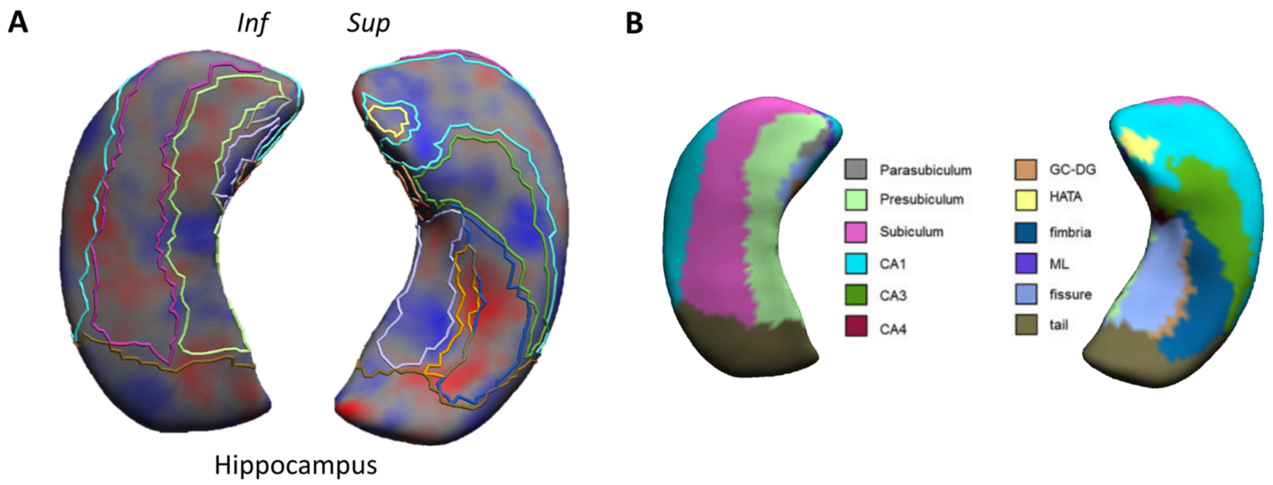

Figure S5A-B. Hippocampal subregions on the ordinal classification maps.
For visualization purposes, we overlaid subregions of the hippocampus according to the FreeSurfer v.6.0 hippocampal subfield atlas (mirrored) onto the effect maps from the ordinal classification model (**A**). The color codes from the hippocampal subregions are shown in (**B**).

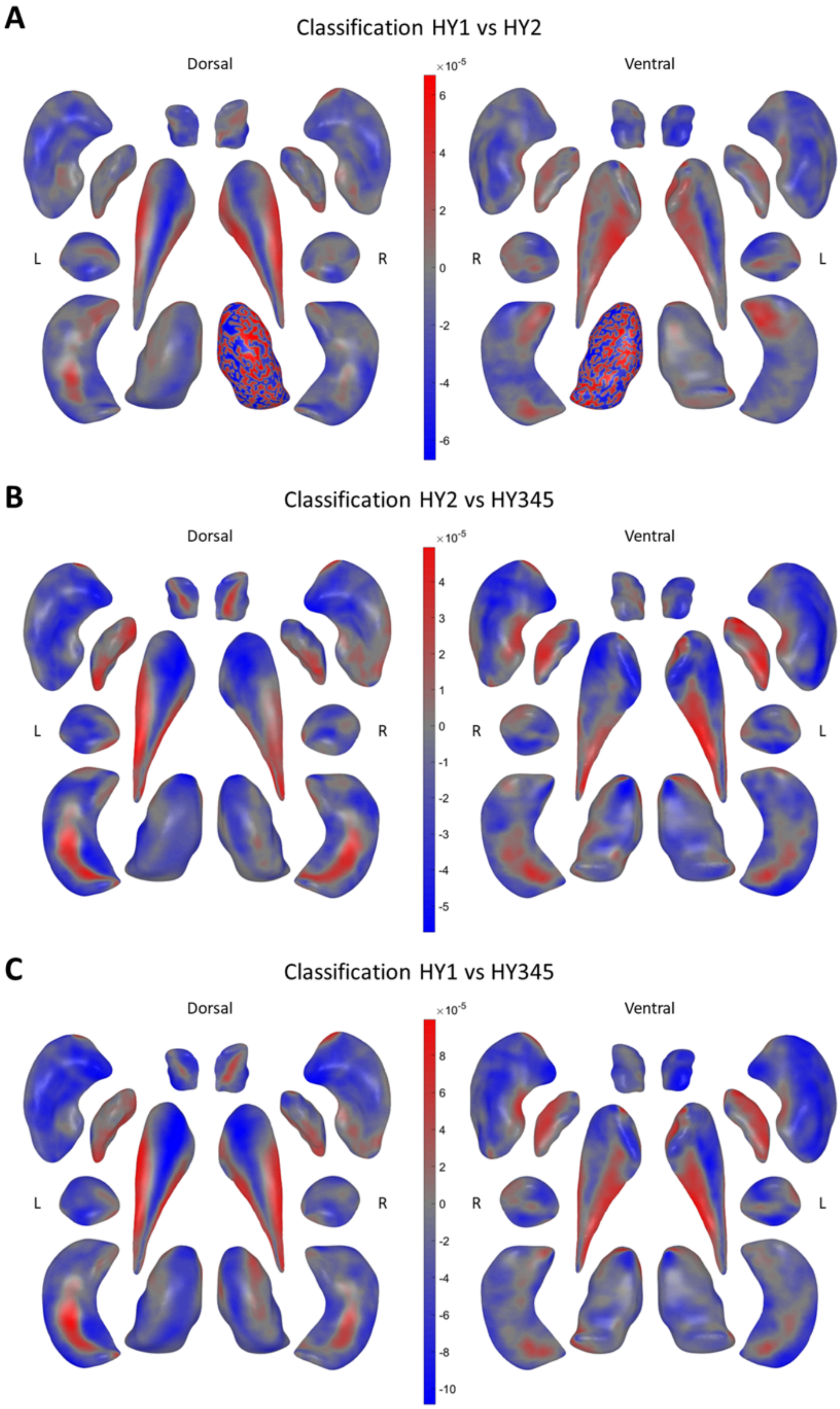

Figure S6A-C. Machine learning binary classification of HY stages versus each other based on the thickness of subcortical structures.

Classification maps of (**A**) HY1 vs HY2, (**B**) HY2 vs HY345, and (**C**) HY1 vs HY345 are shown. The color bars represent learned weights of the classification model, positive values (SD from the learned weights) in red, negative values in blue. More intense colors indicate stronger predictive power of the classification. Note that the right thalamus in panel **A** shows a biologically implausible pattern due to low signal.

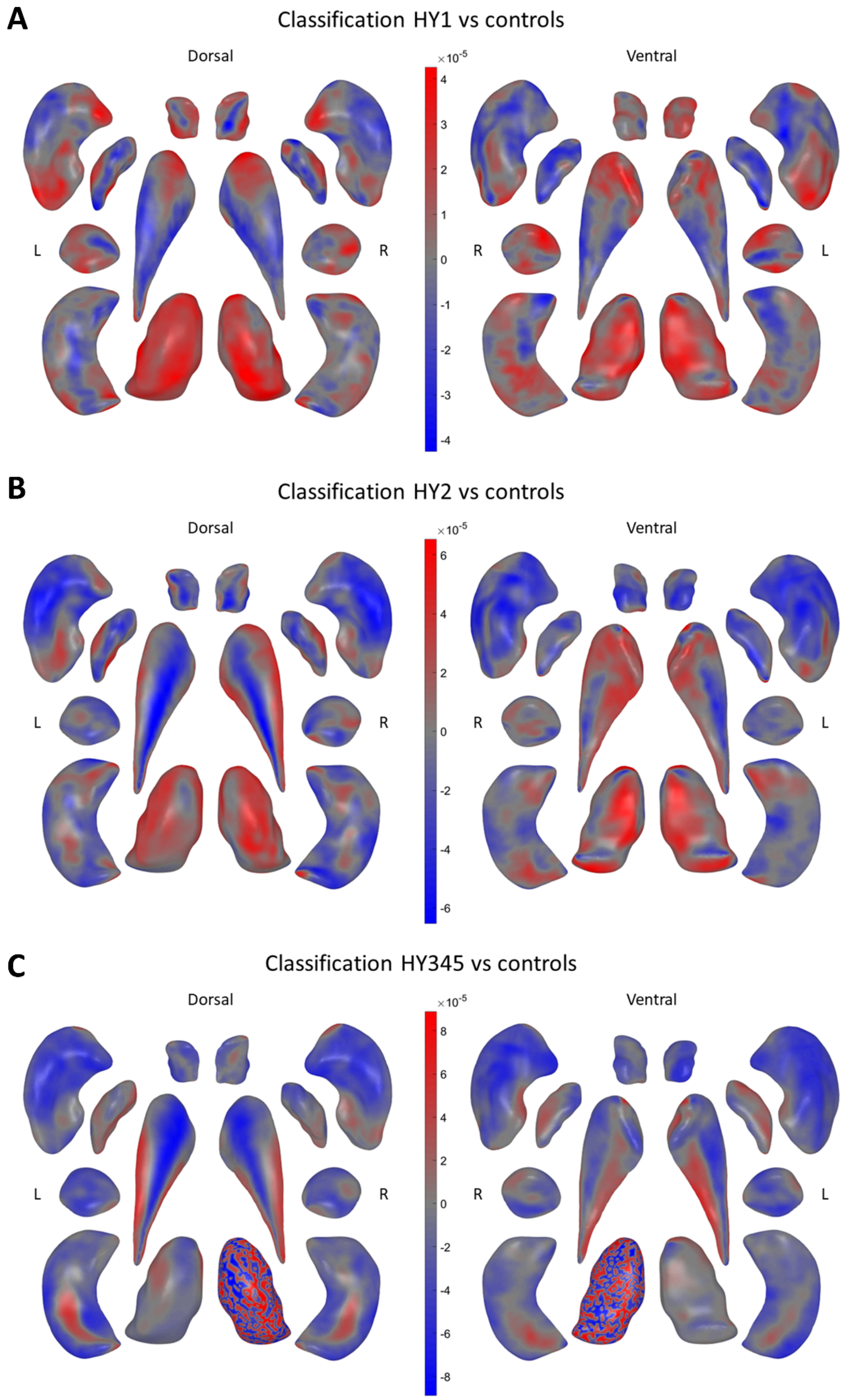

Figure S7A-C. Machine learning: binary classification maps for HY stages versus controls. Binary classification uses vertex-wise thickness information from all subcortical structures. The color bars represent learned weights of the classification model, positive values (SD from the learned weights) in red, negative values in blue. More intense colors indicate stronger predictive power of the classification. Displayed are the results of (**A-C**)

the binary classification of each of the HY stages versus controls. Note that the right thalamus in panel **C** shows a biologically implausible pattern due to low signal.

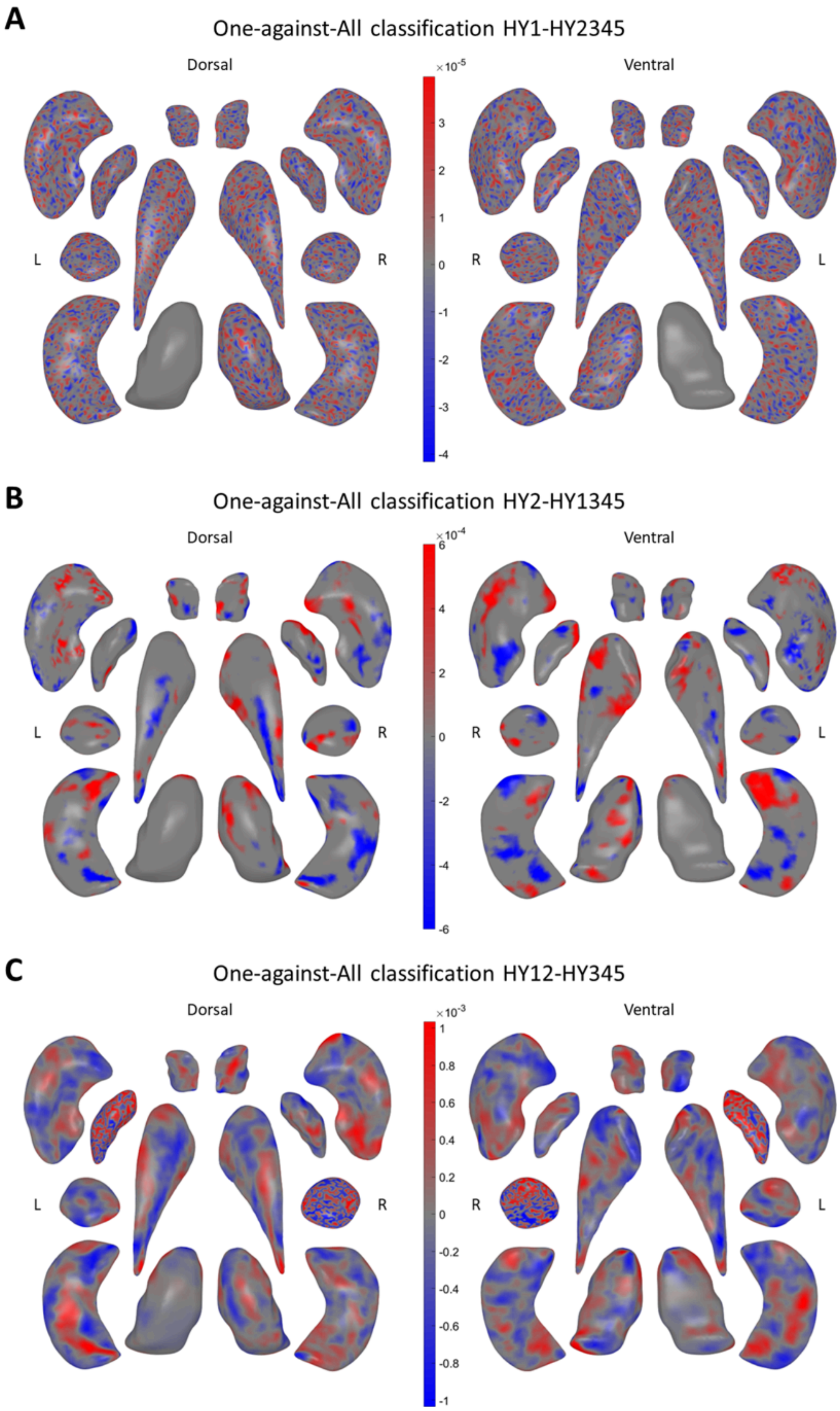

Figure S8A-C. Machine learning: One-against-All classifications of the HY stages.

One-against-All classification maps of (**A**) HY1, (**B**) HY2, and (**C**) HY345 are shown. The color bars represent learned weights of the classification model, positive values (SD from the learned weights) in red, negative values in blue. More intense colors indicate stronger predictive power of the classification. Note that all structures except for the left thalamus in panel **A** and the left globus pallidus and right amygdala in panel **C** show a biologically implausible pattern due to low signal.

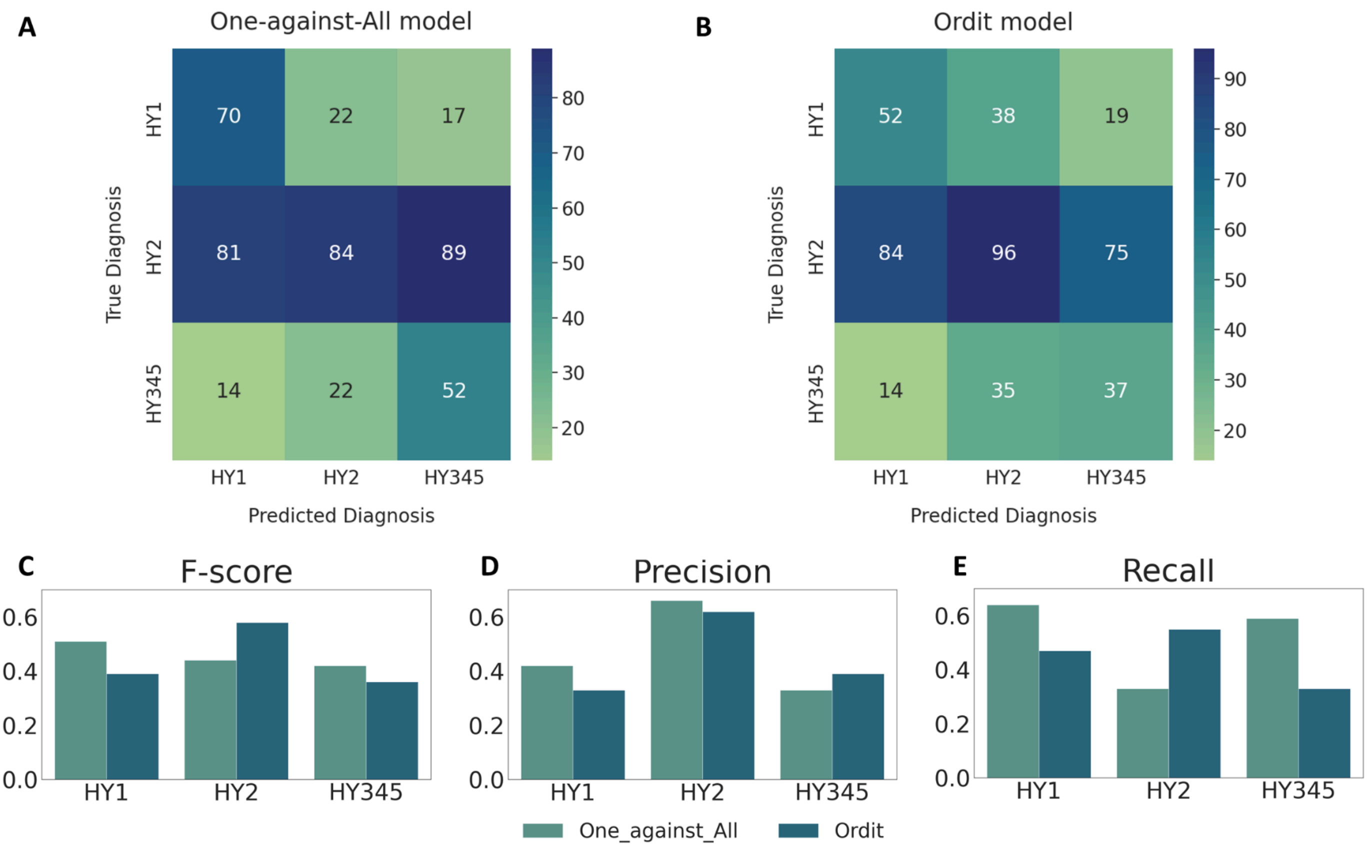

Figure S9A-E. Machine learning: model comparison One-against-All and Ordit.
This figure features a comparison of the multi-task classification performance of One-against-All and Ordit models. It is noteworthy that although the two models' predictive performance scores are comparable (with chance performance of 0.33), Ordit results in a relatively more balanced confusion matrix (**AB**, Table 2) and balanced scores across all classification metrics (**C-E**, Table 2). The collection of anatomical maps representing the One-against-All model is more difficult to interpret than the single Ordit-TVL1 model map, in part because the grouping of different diagnostic categories together does not necessarily reflect the direction of PD-related anatomical change once the disease onset has occurred. Confusion matrices of the (**A**) One-against-All, and (**B**) Ordit models are shown. The ordinal model classifies the plurality of subjects in each HY stage correctly, unlike the One-against-All model. In each row the proportion of misclassified stages is approximately the same due to class-balancing. This is visualized by the diagonal carrying the biggest number. In the lower panels, (**C**) F-score, (**D**) precision, and (**E**) recall are compared between the two models for each HY stage. The corresponding values of performance parameters are provided in Table S13.

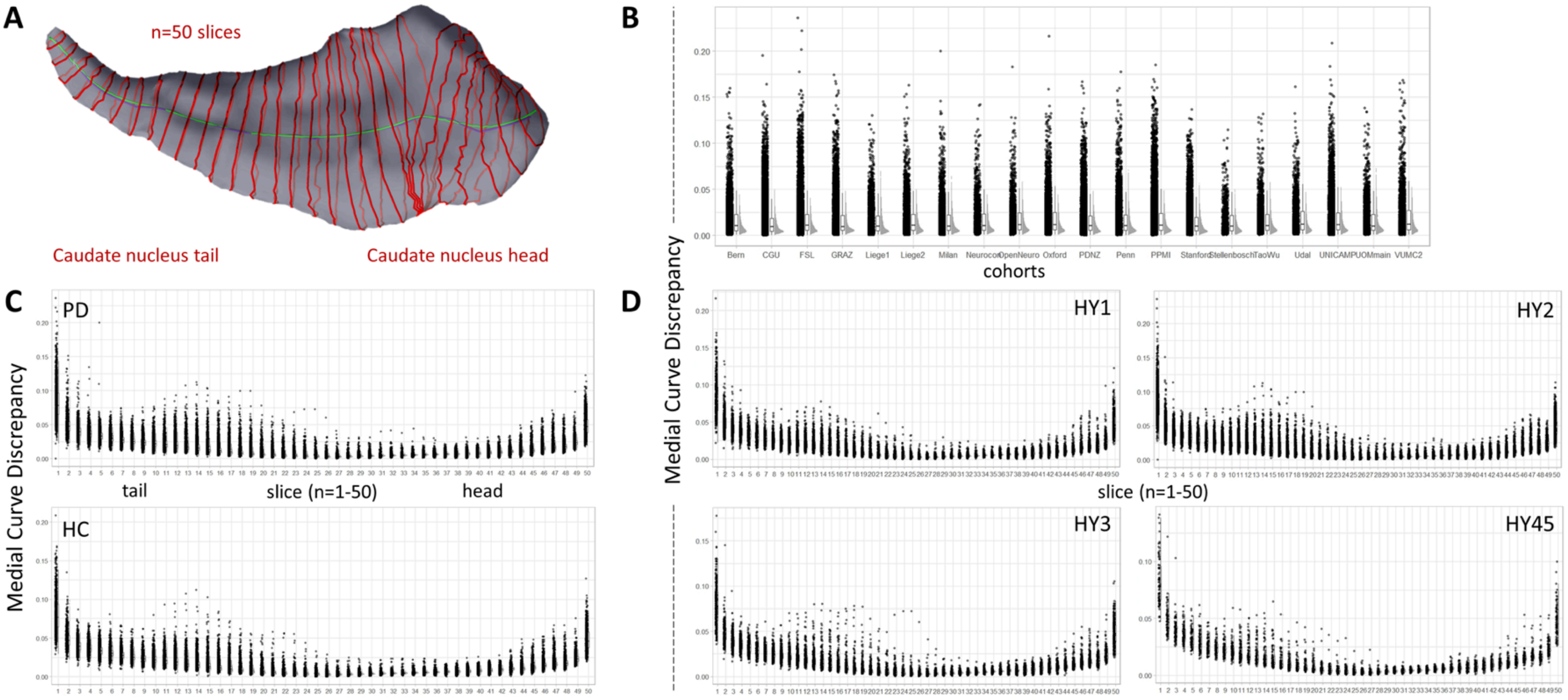

Figure S10A-D. Caudate nucleus medial curve discrepancies along its longitudinal axis.

The pattern of PD vs. controls shape differences in the caudate nucleus revealed by the binary classifiers suggests a "pancaking" effect: an apparent compression along the sagittal axis coupled with an apparent expansion along the axial direction in PD patients relative to controls (Figure 4A, Figure S6A-C). We performed a post hoc analysis to investigate whether this pattern could be caused by some bias in the medial curve. One plausible source of bias is possibly due to the curvature regularization used in fitting the curve. In general, if the overall shape of a region is curved, the medial curve will be pushed towards the concave side of the structure by the regularization. Thus, if the effect of PD were a curving of the caudate nucleus, or if there was bias in segmentation failure of the caudate nucleus in FreeSurfer, this could possibly result in an apparent flattening effect. To check this, we computed the difference between each point on the medial curve and the "true" medial point based on the cross-section of each subject's

caudate nucleus shape at a given location. In this figure, medial curve discrepancies reflect the difference between the medial curve and “true” medial point along the caudate nucleus at 50 slice locations. The medial curve and “true” medial points are shown for (**A**) one subject (1-D “bias” map) and (**B**) did not differ significantly between sites, (**C**) PD and controls, or (**D**) the HY stages. The result was a 1-D “bias” map for each subject (**A**). These results give more credibility to the observed flattening effect in PD indicated by the shape map.

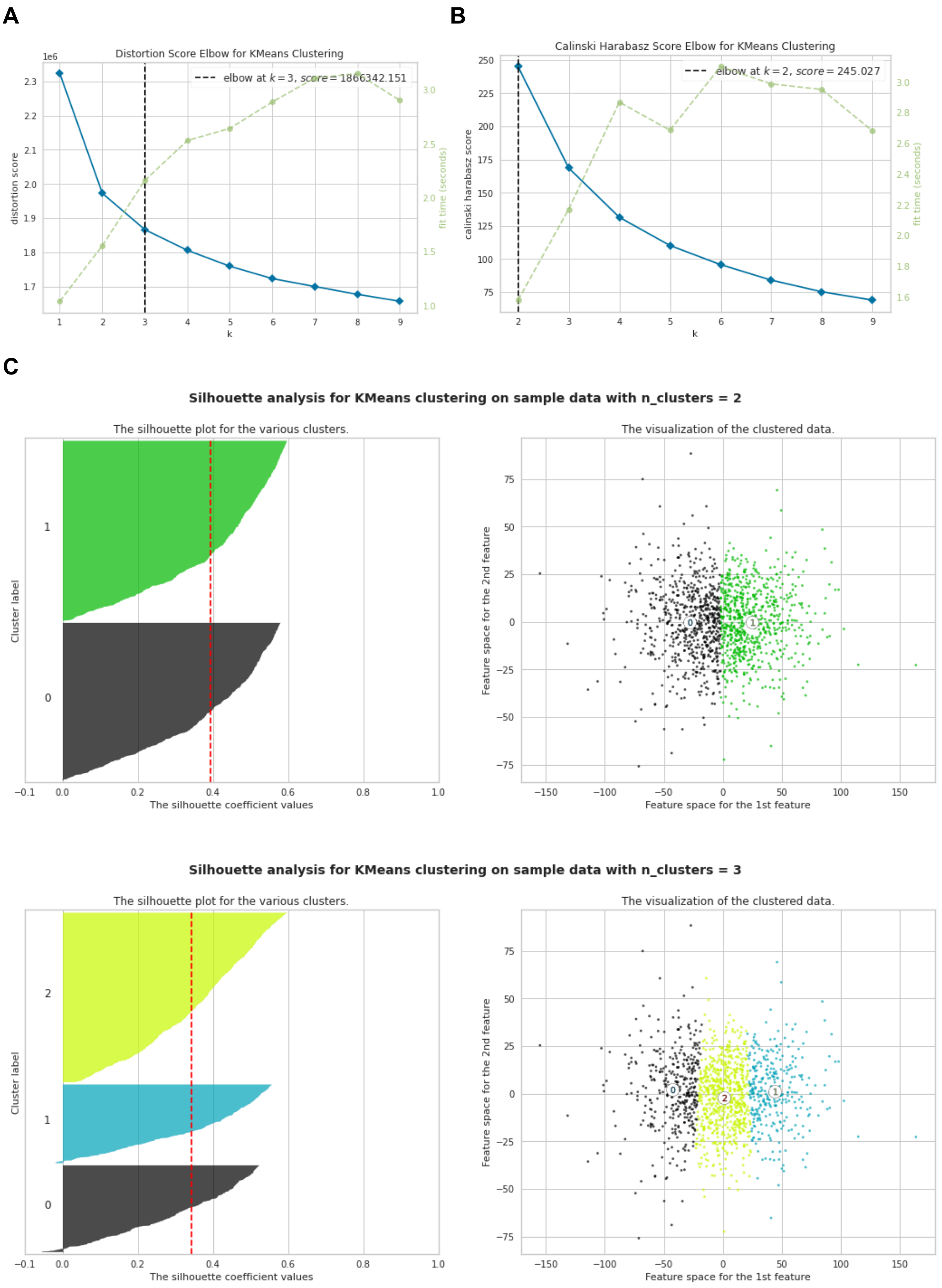

Figure S11A-C. K-means clustering: determining optimal k

We performed k-means clustering (Python package scikit-learn 1.3.2) on the bilateral thalamus vertices in the combined HY1 and HY2 sample set to identify subgroups with shared morphological features. Ordinary least squares means was used to regress out explained variance of age, sex, ICV and cohort. Next, we applied the elbow and

Calinski-Harabasz method in KElbowVisualizer (package yellowbrick 1.5) and silhouette score (scikit-learn) to determine the optimal k clusters. Linear principal component analysis and k-means clustering was performed and identified clusters were statistically compared on clinical characteristics using t-tests and chi-squared tests. (**A**) Elbow method, (**B**) Calinsky-Harabasz method and (**C**) Silhouette method, including scatterplot of clustered data by the first and second principal component. The optimal *k* clusters indicated by the elbow method was two; and by the Calinski-Harabasz index and silhouette score three.

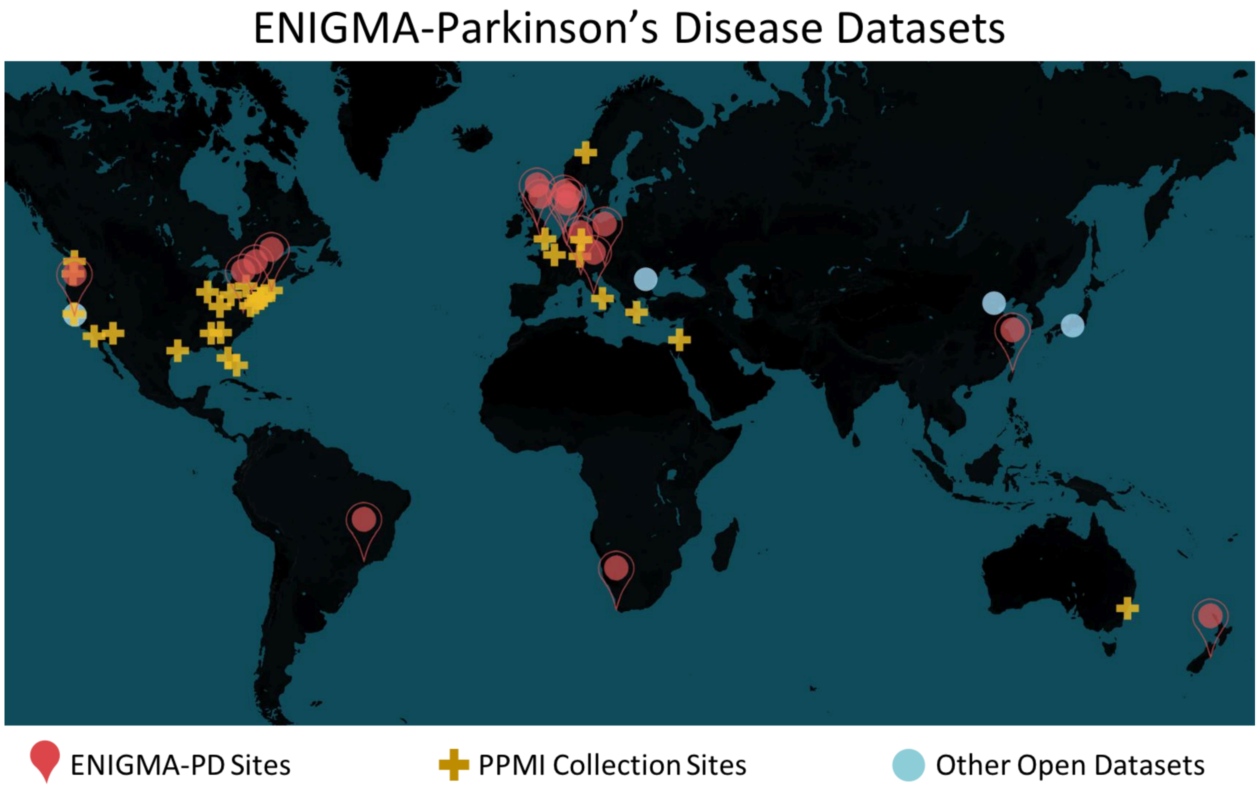

Figure S12. World map of the ENIGMA-Parkinson's Disease Datasets.

Locations of collaborating sites are indicated by red location drops, locations of the Parkinson's Progressive Marker Initiative (PPMI) datasets are indicated by a yellow plus sign, and other open datasets are indicated by a blue circle.

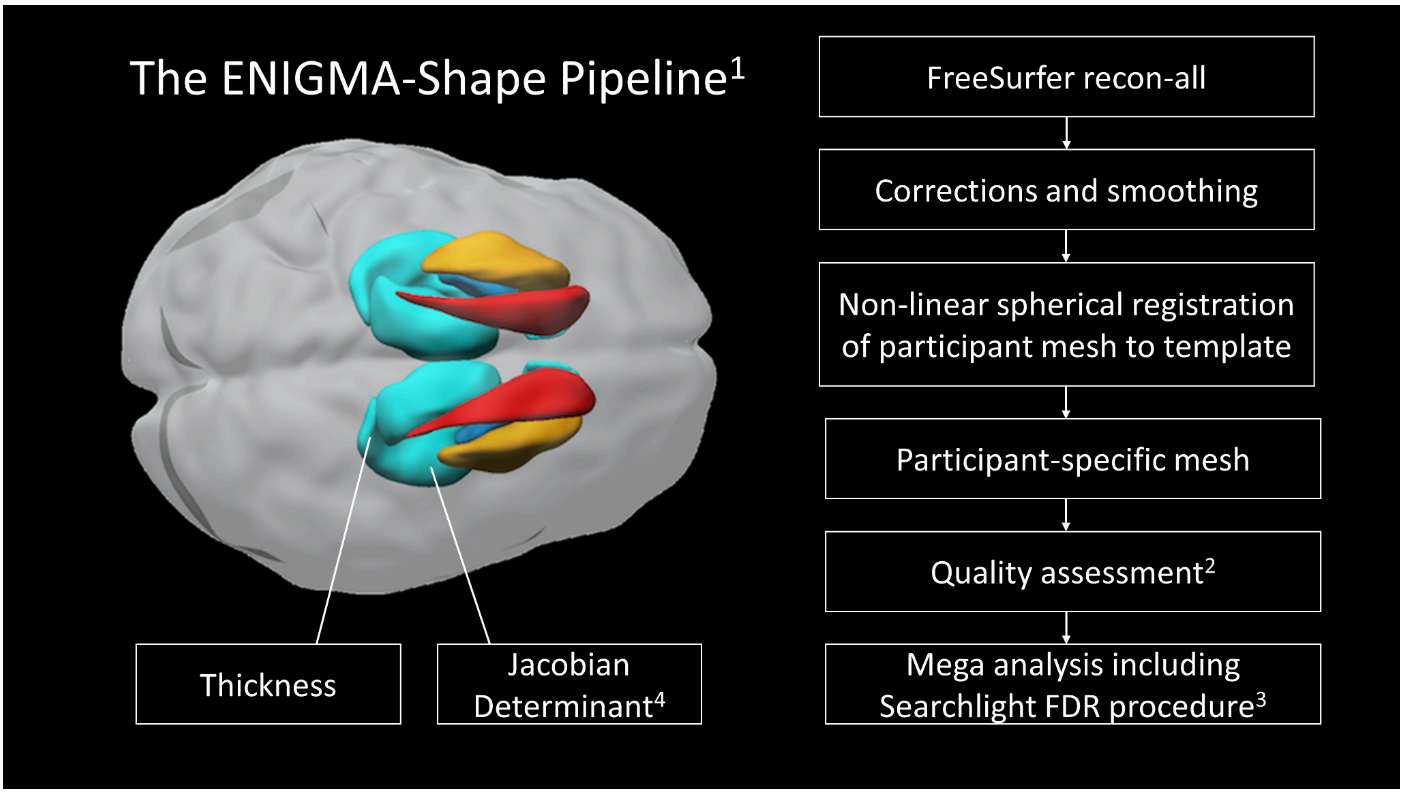

Figure S13. The ENIGMA-Shape Pipeline.

1) The ENIGMA-Shape pipeline uses a template based on subcortical structures from 200 young adults and has been applied to various conditions like major depressive disorder[1], schizophrenia[2], obsessive-compulsive disorder[3], and 22q11.2 Deletion Syndrome[4]. Unlike cortical analysis, subcortical geometries remain relatively stable across ages in healthy adults, primarily varying by volume. Thus, we normalize features in subject-to-template registration to minimize age-related biases in the resulting shape descriptors. The first steps of the ENIGMA-Shape pipeline, including FreeSurfer parcellation[5,6], performed by all source institutes individually. For each participant, we used non-linear spherical registration to align the surface with the template, creating a mesh representing the outer boundaries of the structure. These meshes were then pooled for quality assessment and mega-analysis. 2) Quality assessment entailed visual inspection of the meshes according to the ENIGMA-Shape protocol to assure anatomical accuracy, with exclusion of poorly segmented regions from the analysis. Mean imputation was performed for failed segmentations. We adjusted the overall logistic loss function by the inverse of the diagnostic or staging label frequency, to account for the imbalance in PD/control and HY stage group size. 3) In previous work[2], we modified the searchlight FDR procedure[7,8] for global application across all structures in each linear model. Distances between vertices were defined as the Euclidean distance, with those between different structures set to infinity. The correction is more conservative than one that accounts for correlations between different regions but less conservative than the original FDR approach[9], which ignores spatial correlation. 4) In this study, we focused on thickness as a shape morphometry measure as it offers intuitive interpretability in the context of neurodegeneration. We also analyzed the logarithm of the Jacobian determinant (Figure S14A-H), which reflects the ratio of two local surface areas: a surface patch on the participant and template surface[10,11]. Log-Jacobian values below 1 indicate surface contraction and values above 1 indicate expansion. The two measures of shape morphometry are considered complementary to each other (Figure S15A-C).

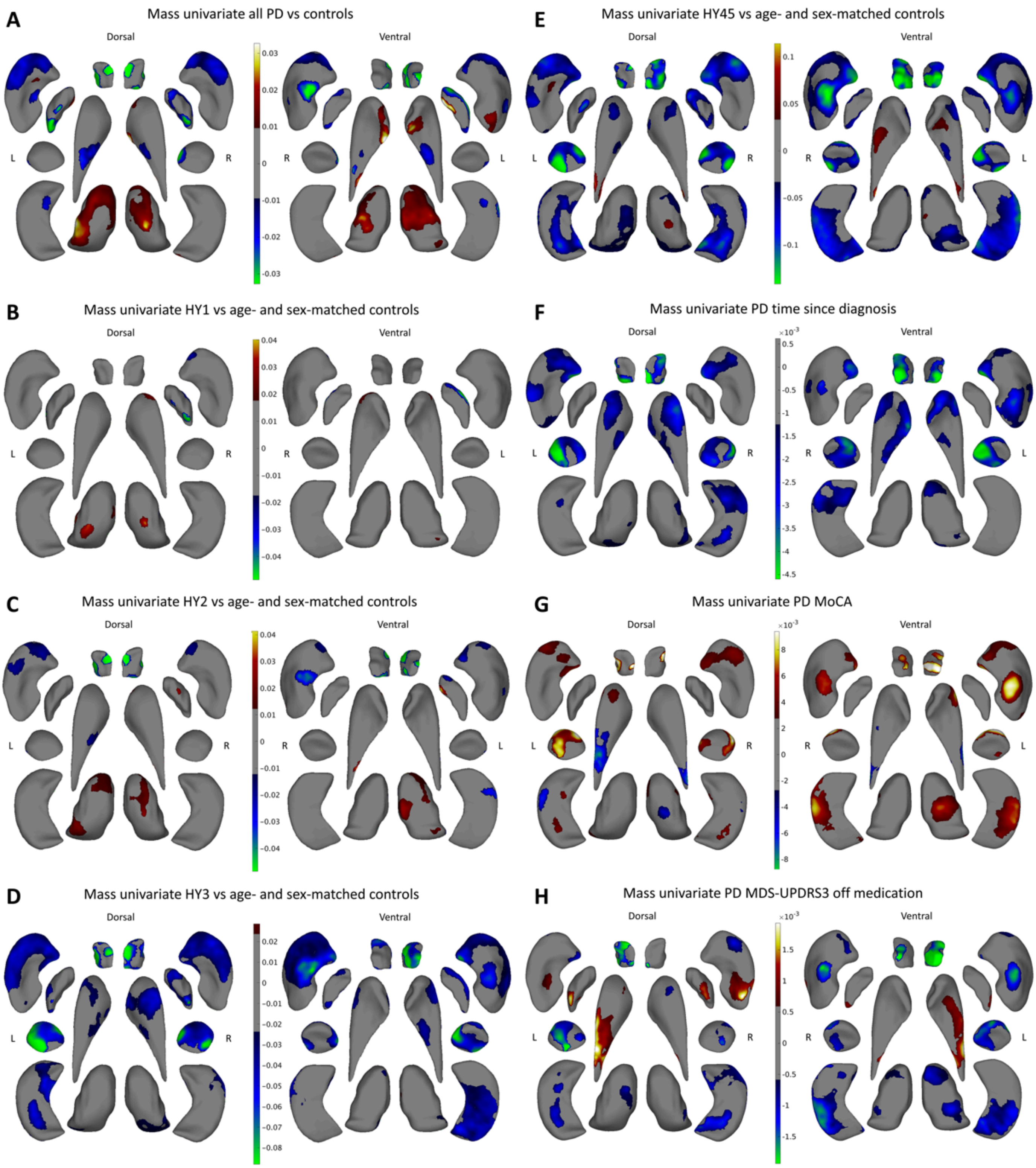

Figure S14A-H. Mass univariate analysis: significant vertex-wise differences in Jacobian determinant between people with PD and controls.

The patterns observed in the thickness analyses partly aligned with those observed in the Jacobian determinant analyses. The Jacobian determinant results are also presented in Table S13A-H. Conceptually, the Jacobian determinant and thickness differ in interpretability; whereas the Jacobian is an indirect reflection of surface expansion or contraction, the thickness values directly represent the absolute difference in tissue thickness. A comparison of both measures of shape deformation is visualized in Figure S15A-C. Effect maps are projected onto the subcortical regions showing the (**A**) PD versus controls, and (**B-E**) subgroup comparisons, and (**F-H**) correlations with clinical

markers. Positive b-values indicate that regions are thicker, negative b-values indicate that regions are thinner in PD compared to controls. (**B-E**) All HY groups are sex- and age-matched. (**A-H**) The models are corrected for intracranial volume and cohort in all panels, and additionally corrected for age and sex in panel **H**.

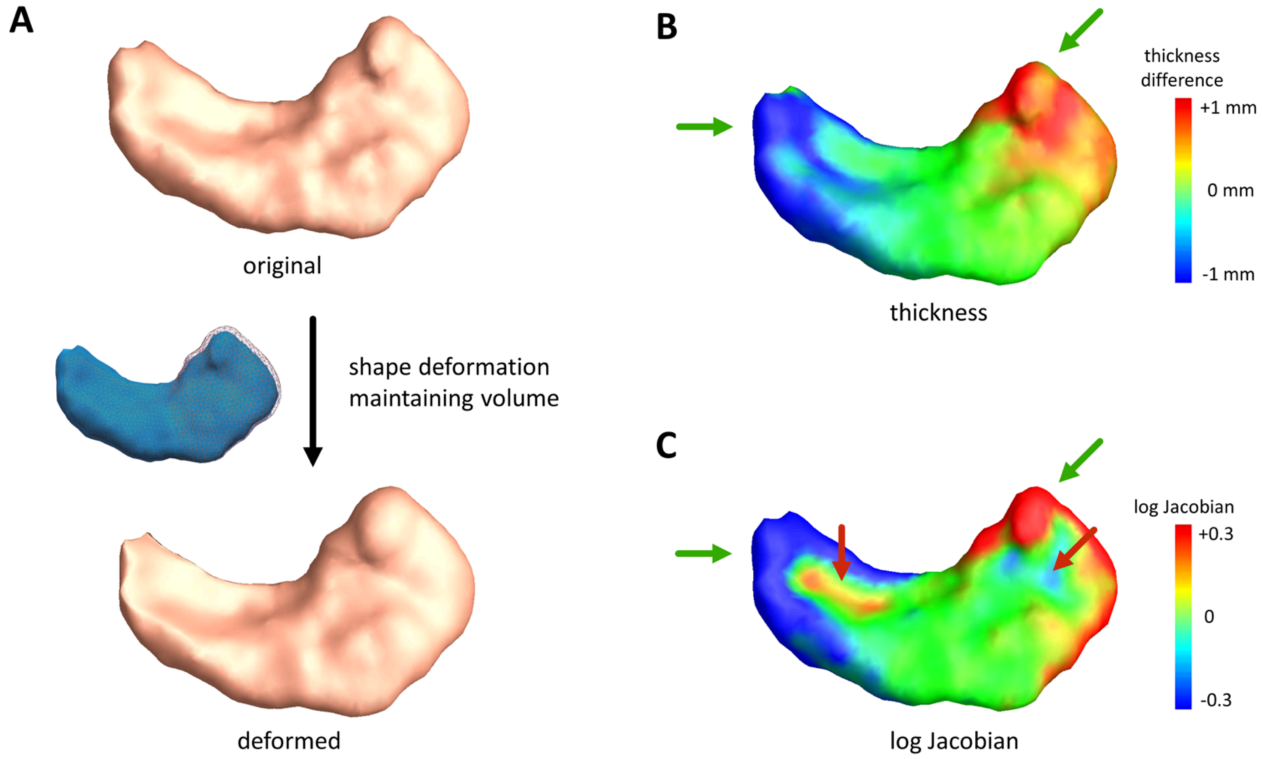

Figure S15A-C. Comparison of log Jacobian versus thickness effects.
Comparing the effects of a volume-preserving deformation (**A**) on thickness (**B**) and log Jacobian (**C**) measures in a hippocampal surface model. Notably, the direction of effect is concordant between the two measures in areas of positive curvature and discordant in areas of negative curvature. Another example is to imagine a stretching of the hippocampus along the main axis. The thickness would remain unchanged, while the jacobian would increase. The arrows in panels **B** and **C** highlight similarities (green) and differences (red) between the two measures of shape deformation.

Supplementary References (cited in this supplement)

1. Ho, T. C. *et al.* Subcortical shape alterations in major depressive disorder: Findings from the ENIGMA major depressive disorder working group. *Hum. Brain Mapp.* **43**, 341–351 (2022).
2. Gutman, B. A. *et al.* A meta-analysis of deep brain structural shape and asymmetry abnormalities in 2,833 individuals with schizophrenia compared with 3,929 healthy volunteers via the ENIGMA Consortium. *Hum. Brain Mapp.* **43**, 352–372 (2022).
3. Fouche, J.-P. *et al.* Shape analysis of subcortical structures in obsessive-compulsive disorder and the relationship with comorbid anxiety, depression, and medication use: A meta-analysis by the OCD Brain Imaging Consortium. *Brain Behav.* **12**, e2755 (2022).
4. Ching, C. R. K. *et al.* Mapping Subcortical Brain Alterations in 22q11.2 Deletion Syndrome: Effects of Deletion Size and Convergence With Idiopathic Neuropsychiatric Illness. *Am. J. Psychiatry* **177**, 589–600 (2020).
5. Dale, A. M., Fischl, B. & Sereno, M. I. Cortical surface-based analysis. I. Segmentation and surface reconstruction. *Neuroimage* **9**, 179–194 (1999).
6. Gutman, B. A., Madsen, S. K., Toga, A. W. & Thompson, P. M. A Family of Fast Spherical Registration Algorithms for Cortical Shapes. in *Multimodal Brain Image Analysis* 246–257 (Springer International Publishing, 2013).
7. Kriegeskorte, N., Goebel, R. & Bandettini, P. Information-based functional brain mapping. *Proc. Natl. Acad. Sci. U. S. A.* **103**, 3863–3868 (2006).
8. Langers, D. R. M., Jansen, J. F. A. & Backes, W. H. Enhanced signal detection in neuroimaging by means of regional control of the global false discovery rate. *Neuroimage* **38**, 43–56 (2007).
9. Hochberg, Y. & Benjamini, Y. More powerful procedures for multiple significance testing. *Stat. Med.* **9**, 811–818 (1990).
10. Gutman, B. A. *et al.* Medial Demons Registration Localizes The Degree of Genetic Influence Over Subcortical Shape Variability: An N= 1480 Meta-Analysis. *Proc. IEEE Int. Symp. Biomed. Imaging* **2015**, 1402–1406 (2015).
11. Wang *et al.* Surface-based TBM boosts power to detect disease effects on the brain: an N= 804 ADNI study. *Neuroimage* (2011).
